# Computational origins of shape perception

**DOI:** 10.1371/journal.pcbi.1013674

**Published:** 2025-12-15

**Authors:** Lalit Pandey, Samantha M. W. Wood, Justin N. Wood

**Affiliations:** 1 Informatics Department, Indiana University Bloomington, Bloomington, Indiana, United States of America; 2 Cognitive Science Program, Indiana University Bloomington, Bloomington, Indiana, United States of America; 3 Department of Neuroscience, Indiana University Bloomington, Bloomington, Indiana, United States of America; 4 Center for the Integrated Study of Animal Behavior, Indiana University Bloomington, Bloomington, Indiana, United States of America; Technical University of Denmark Department of Health Technology: Danmarks Tekniske Universitet Institut for Sundhedsteknologi, DENMARK

## Abstract

Shape perception is central to human vision, but its developmental origins remain unknown. Here we show that shape perception develops from three ingredients: (1) generic fitting systems, (2) embodied visual experiences, and (3) biologically plausible sensors. We first show that generic fitting models (transformers) trained on embodied visual experiences change from color-based to shape-based visual systems. We then perform *in silico* controlled-rearing experiments to determine what causes this developmental change. We find that view diversity—experiencing many views of the same object—produces shape perception. For embodied agents, view diversity comes for free: by moving through the world, agents acquire diverse temporally linked views, explaining how and why animals develop shape perception so rapidly. But when view diversity is restricted, by limiting where an agent can look or move, shape perception fails to develop. Finally, we show that retinas naturally transform images in ways that enhance shape learning, providing a biologically plausible substitute for artificial image augmentations. Together, our results support generic fitting theories of brain development and provide a template for building human-like shape perception in machines.

## Author summary

Understanding the origins of shape perception is a long-standing challenge in psychology and neuroscience. We bring together methods from developmental psychology, computational neuroscience, virtual reality, and artificial intelligence to characterize the learning mechanisms, experiences, and sensors for developing shape perception. We find that shape perception develops from three ingredients: (1) *generic fitting systems* that operate by the same general fitting principles as evolution, (2) *embodied visual experiences* that include diverse temporally linked views of objects, and (3) *biologically plausible sensors* that reformat raw visual input like the human retina. When artificial agents are equipped with generic fitting systems (brains) and human-like artificial retinas (eyes), the agents automatically develop a shape-centric visual system that categorizes novel objects by shape. Our results provide an image-computable origin story for shape perception.

## Introduction

Shape perception is central to human vision [1–4]. We perceive the world in terms of bounded, cohesive objects that maintain their shape across space and time [5]. We prioritize shape over other cues (e.g., size, color, texture) when recognizing and reasoning about objects [6–9]. Shape perception is present early in development [10] (in some species, within days after birth [11,12]) and influences language learning, with toddlers showing robust shape biases when generalizing words to new objects [13]. Shape perception is also found across the animal kingdom, including in monkeys [14], rodents [15], birds [16], and insects [17]. Across developmental and evolutionary timescales, shape perception is a universal feature of biological vision.

To date, however, the origins of shape perception are unknown. Is shape perception innate or learned? If shape perception is learned, what ingredients (learning mechanisms and experiences) underlie learning? Nativists propose that shape perception depends on innate (unlearned) primitives [18,19]. For example, Gestalt psychologists argued that principles of visual organization are innate, with brains prewired to organize sensory input according to certain rules [18]. Empiricists, in contrast, propose that shape perception is learned. Evidence for shape learning comes from perceptual learning effects in human adults [20–25], object learning studies in children [26,27], and neurophysiological studies showing that shape perception substrates in brains are plastic, adapting to the spatiotemporal statistics of raw visual experience [28,29]. Importantly, both camps agree that genes produce priors for learning, but they disagree about the nature of those priors: nativists argue that initial priors have domain-specific content (e.g., priors for objects, shape, space, number), whereas empiricists argue that initial priors are domain general (e.g., domain-specific knowledge of objects, shape, space, and number is learned from experience).

Both accounts (innate vs. learned) are well supported by evidence, with nativists emphasizing that object perception is innate given its rapid development in human babies [19] and newborn animals [12], and empiricists countering that shape perception is learnable, since deep neural networks (DNNs) can learn shape perception without hardcoded knowledge of objects or space [30–32]. However, no prior theories have formalized both the *mechanisms* and *experiences* that cause shape perception to develop. Nor do prior theories provide sufficient guidance for building image-computable models that develop shape perception in the same environments as animals, including both natural environments and the impoverished environments faced by newborn animals in controlled-rearing studies [11,12]. Finally, prior computational modeling studies have largely ignored the role that visual *sensors* (eyes) play in the development of shape perception. If a model developed in the same environment as a newborn, what mechanisms, experiences, and sensors would it need to develop shape perception?

One possibility is the model would need learning processes that operate by the same general fitting principles as evolution [33–35]. In evolution, genetic variation generates a range of possibilities, and selection filters those possibilities based on fitness. The products of evolution (peaks on the fitness landscape) are animal species. Learning can also be conceptualized as a variation + selection fitting process, in which prenatal development generates a range of variation (trillions of connection weights in prenatal brains) and experience selects (strengthens/weakens) those connection weights to produce adaptive behavior. Under this view, mental skills are the products of generic fitting processes occurring during an individual’s lifetime. Thus, shape perception might be the product of a ‘mini-evolution’ in individual organisms, where brains start from scratch (no innate knowledge of shape) and gradually develop shape perception as brains fit to the organism’s prenatal and postnatal environment [36,37].

Specifically, we use “generic fitting systems” to refer to over-parameterized, domain-general models that learn task-relevant structure by directly fitting large numbers of adjustable parameters to densely sampled, structured experience, rather than by relying on hardcoded, domain-specific priors (e.g., for objects, shape, space, number). In generic fitting systems, intelligence emerges through iterative optimization of an objective over high-dimensional embeddings, without hardcoded domain-specific priors to guide the fitting process. By this definition, transformer models qualify as generic (no hardcoded domain-specific priors), whereas CNN models do not, since convolution and weight sharing impose strong spatial priors on learning. Our use of “generic fitting systems” is intentionally parallel to the evolutionary case: evolution and domain-general models are members of the same family of direct-fit optimization processes: iterative, objective-guided fitting that leverages the structure of the environment, without encoding domain-specific priors in advance [35].

Here, we directly test the hypothesis that shape perception is the product of generic fitting. To do so, we use transformers: generic DNNs that learn from variation + selection fitting. The models started with random weights and large numbers of connections (variation), followed by gradual adjustment of those weights to optimize the learning objective (selection). Selection was determined by a biologically plausible learning objective—unsupervised temporal learning—that leveraged time as a teaching signal, akin to temporal learning in brains [22,38–42].

The model (Vision Transformer with Contrastive Learning through Time, ViT-CoT) had a Vision Transformer architecture and self-supervised Contrastive Learning Through Time objective [43] that pushed together embeddings of video frames that temporally co-occurred, while separating embeddings that did not co-occur [44]. ViT-CoT is a generic fitting model that starts with no hardcoded domain-specific knowledge (untrained state), then gradually adapts its representational space during training to fit the spatiotemporal data in raw visual experiences. To ensure that the fitting models had the same training data as biological visual systems, we trained the models with embodied visual experiences from human adults and newborn animals.

We also systematically manipulated the number of transformer blocks and attention heads to assess the robustness of our findings across model capacities. Specifically, we varied the size of the fitting models by systematically adding transformer blocks and attention heads, resulting in four different sizes (1H, 3H, 6H, and 9H; Methods). For example, the ViT-CoT(3H) model had three attention heads and three transformer blocks. This approach helps ensure that the emergence of shape sensitivity is not a specific artifact of a particular architecture size. Rather than aiming for direct biological plausibility, our goal was to test whether the computational principles underlying shape perception generalize across architectures of varying capacity. By varying architecture size, we could also measure whether fitting models need a minimum number of adjustable parameters to develop shape perception from embodied data streams.

In the following sections, we first evaluated untrained fitting models and found that their representational spaces are color-based, organizing novel objects by color rather than shape. We then trained the models on first-person visual experiences from human adults and found that the models spontaneously transformed into shape-based visual systems. Thus, generic fitting systems are sufficient to develop shape perception.

To isolate the particular experiences that drive shape learning, we performed *in silico* controlled-rearing experiments on fitting models, systematically manipulating the visual experiences used to train the models. Despite having no hardcoded shape knowledge, generic fitting models developed shape perception when trained in the same impoverished worlds as newborn animals in controlled-rearing experiments (i.e., environments with a single object).

We make sense of this surprising result by showing that the relevant experiences for developing shape perception are not *objects*, but *views*. When embodied agents move, they see diverse temporally linked views of the world. Fitting models leverage these diverse views to learn shape perception. When we reduced view diversity, fitting models failed to learn shape perception.

Finally, we show that the human retina, with its unique distribution of rods and cones, transforms raw visual experience to produce the kind of view diversity needed to learn shape perception. Thus, we show that shape perception develops from three ingredients: (1) generic fitting systems, (2) embodied visual experiences, and (3) biologically plausible sensors.

## Results

### Evaluating representational spaces of fitting models

**Fig 1:**
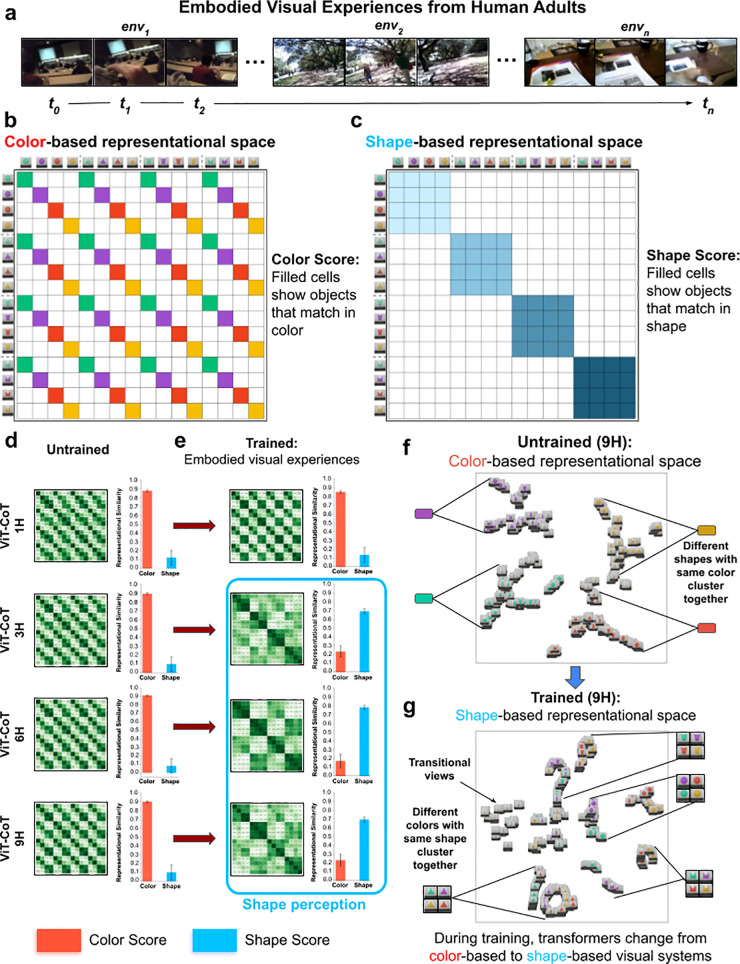
Training generic fitting models on embodied visual experiences. (a) First-person visual experiences collected from human adults, wearing head-mounted cameras. The videos were recorded in natural environments, with large numbers of objects, people, and places. (b-e) We used RDMs to evaluate whether generic fitting models have color-based versus shape-based representational spaces. (b) Color scores were computed by averaging across RDM cells where objects matched in color (filled cells in Panel b). (c) Shape scores were computed by averaging across RDM cells where objects matched in shape (filled cells in Panel c). (d) Untrained fitting models have color-based representational spaces, as shown in the RDMs (*left*) and color/shape scores (*right*). (e) Trained fitting models developed shape-based representational spaces. The one exception was the smallest (1H) model, which failed to develop shape perception. (f) t-SNE visualizations showed that untrained generic fitting models group objects using color, whereas (g) trained generic fitting models group objects based on shape. The images in the t-SNE visualization correspond to frames captured as the object rotated in the simulation. Error bars denote standard error for each model across the color cells and shape cells shown in Fig 1b, c.

Our goal was to characterize the origins of shape perception, which required testing whether models categorize objects by shape rather than other features (e.g., color). To test the models, we generated a stimuli set containing 16 objects, composed of all combinations of four shapes and four colors (Fig 1b). If a model’s representational space is color-based, then objects of the same color (but different shape) will group together (Fig 1b and 1d); conversely, if a model’s representational space is shape-based, then objects of the same shape (but different colors) will group together (Fig 1c and 1e).

We analyzed the representational spaces of models in three ways. First, we used representational dissimilarity matrices (RDMs) [45]. The RDMs show the distances between representations of image pairs in a model’s embedding space. With RDMs, we can visualize whether a model builds more similar representations of objects that match in color (Fig 1b and 1d) versus shape (Fig 1c and 1e). Second, to quantify whether models were color-based versus shape-based, we computed the average representational similarity between objects that matched in color (*color score,* Fig 1b) versus shape (*shape score*, Fig 1c). We then compared the color and shape scores across models. Third, to measure how objects group within the representational spaces of the models, we used t-SNE plots [46]. t-SNE plots visualize the model’s representational space by mapping the high-dimensional embedding of each object into a 2D space, while preserving the local structure of the data. These plots clearly show whether a model’s representational space clusters objects by color (Fig 1f) versus shape (Fig 1g). For all experiments, the color scores, shape scores, and statistical analyses are reported in Methods.

### Training generic fitting models on visual experiences from human adults

We first measured whether untrained fitting models have color-based or shape-based representational spaces. We found that untrained models have robust color-based representational spaces (Fig 1d). Untrained models group objects by color, not shape (Fig 1f). None of the untrained architecture sizes showed evidence for shape perception, indicating that these generic fitting models lack hardcoded knowledge about shape.

Next, we explored whether generic fitting models develop shape perception when trained on egocentric visual experiences from human adults (Fig 1a). We used the UT Ego dataset [47], which includes longitudinal egocentric videos of humans performing a range of activities in many environments. During training, each model used contrastive learning through time, where the images “brought together” in the representational space were data-augmented versions of images that appeared in the same temporal window (three images, ~ 300 ms). Like most top-performing computer vision models [48–50], our models also increased view diversity by adding hardcoded image augmentations during training. Our models used five image augmentations that are common in contrastive learning: gaussian blur (50% of images), grayscale (20% of images), random resized crop (100% of images), random horizontal flip (50% of images), and color jitter (80% of images). In our final experiments, we return to artificial image augmentations and directly measure their impact on shape perception; ultimately, we propose that artificial retinas can replace artificial image augmentations to permit the development of shape perception in generic fitting systems.

During training, the three largest models (3H, 6H, 9H) spontaneously developed shape perception (Fig 1e). The exception was the smallest (1H) model, which learned partial shape perception. Thus, trained generic fitting models group objects by shape, not color (Fig 1g), provided that the models are sufficiently large. To validate this conclusion, we tested the models with different stimuli (new simple shapes and colors) and replicated the original pattern (S1 Fig).

**Fig 2:**
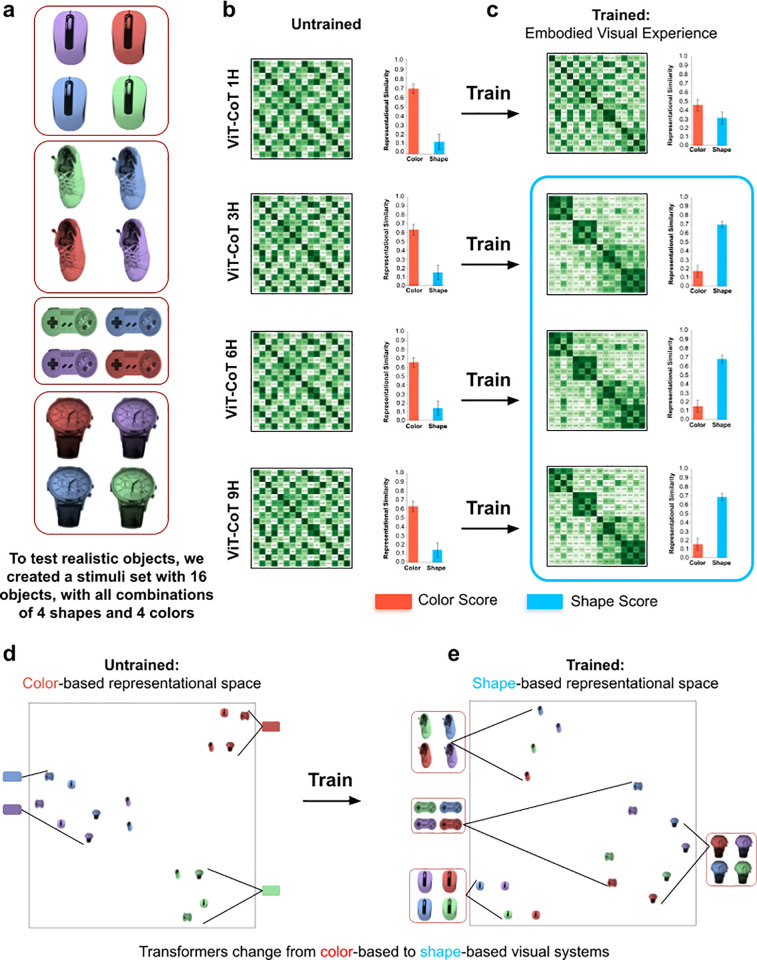
Realistic Objects. (a) To test whether our results generalize to realistic objects, we tested the models on objects with more complex features. (b) Untrained transformers had color-based representational spaces, as shown in the RDMs (*left*) and color/shape scores (*right*). (c) Trained transformers developed shape perception, grouping objects by shape rather than color. The exception was the smallest (1H) model, which developed partial shape perception. (d) t-SNE visualizations showed that untrained models group objects based on color, whereas (e) trained models group objects based on shape. These models were trained on the human adult data from Experiment 1. Error bars denote standard error for each model across the color cells and shape cells shown in Fig 1b, c.

Since the test objects used above were simple geometric shapes, we also created a new stimuli set with more complex and realistic objects (Fig 2a). Again, untrained models had robust color-based representational spaces (Fig 2b), grouping objects by color, not shape (Fig 2d). Conversely, after training, the three largest models (3H, 6H, 9H) developed shape perception (Fig 2c), grouping objects by shape, not color (Fig 2e). Thus, our results generalize to more realistic objects.

These findings build on prior work showing that generic fitting models can learn object perception from the egocentric visual experiences of humans [31,32]. Our experiments extend these studies by showing that generic fitting models transform from color-based to shape-based visual systems during training. But, like prior work, our training data was uncontrolled, preventing deep understanding of the role of experience in developing shape perception. Which particular experiences drive learning of shape perception?

We answer this question by turning to controlled rearing, a classic method from psychology and neuroscience for characterizing the role of experience in learning and development [51–54]. We performed *in silico* controlled-rearing experiments on generic fitting models to determine the role of experience in the development of shape perception.

### Controlled-rearing experiments on generic fitting models

**Fig 3:**
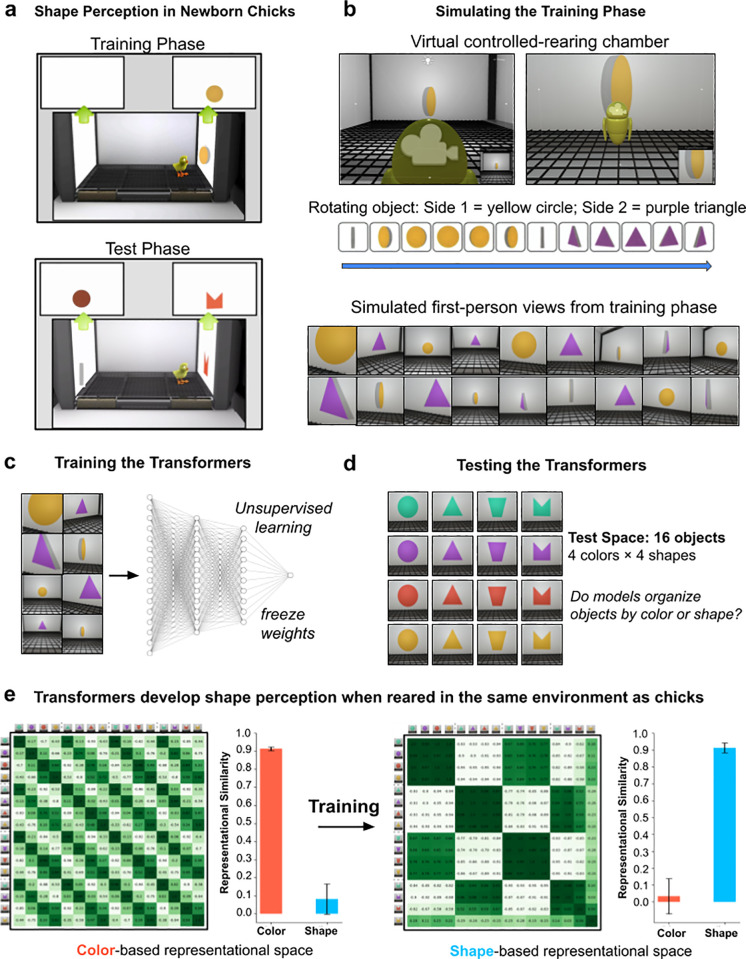
Comparing learning across newborn animals and generic fitting models. (a) Wood^11^ showed that newborn chicks develop shape perception when reared with a single object. During the training phase, the chick’s environment contained a single object. During the test phase, the chambers measured whether the chicks developed shape perception. (b) To simulate the chick study, we created virtual animal chambers and simulated the first-person visual experiences available to the chicks during the training phase. As in the chick study, the virtual chambers presented one object that rotated continuously, revealing a different color and shape on each of its two sides. (c) We trained generic fitting models (transformers) using the simulated visual experiences from the virtual chamber. (d) To test the models, we simulated the visual experiences available to the chicks during the test phase. (e) Visualization of the representation spaces of untrained versus trained fitting models. Untrained fitting models (ViT-CoT 6H shown here) organize objects based on color, whereas models trained on the visual experiences of newborn chicks learned to organize objects based on shape. Error bars denote standard error for each model across the color cells and shape cells shown in Fig 1b, c.

We first tested whether generic fitting models develop shape perception when trained on simulated visual experiences from newborn chicks (Fig 3). We focused on chicks because the strongest evidence that shape perception is innate comes from studies of chicks, who have robust shape perception soon after hatching [55,56]. In fact, chicks develop shape perception even when raised in impoverished environments containing *a single object* [11,12]. These findings present a strong challenge to learning theories because it seems unlikely that shape perception could be learned from visual experience of a single object, especially for generic fitting systems that start with no hardcoded knowledge about objects, shape, or space.

However, recent studies suggest that shape perception might be learnable from the visual experiences available to newborn chicks [44,57]. Pandey and colleagues simulated the first-person views of chicks in controlled-rearing studies and found that generic fitting models trained on those simulated views develop the same view-invariant object recognition skills as chicks. Thus, fitting models can learn to recognize familiar objects across novel views. Do generic fitting models also learn to categorize *new* objects by shape, consistent with shape-centric vision? If so, then models trained in the same visual worlds as chicks should spontaneously learn to categorize novel objects by shape, rather than color.

**Fig 4:**
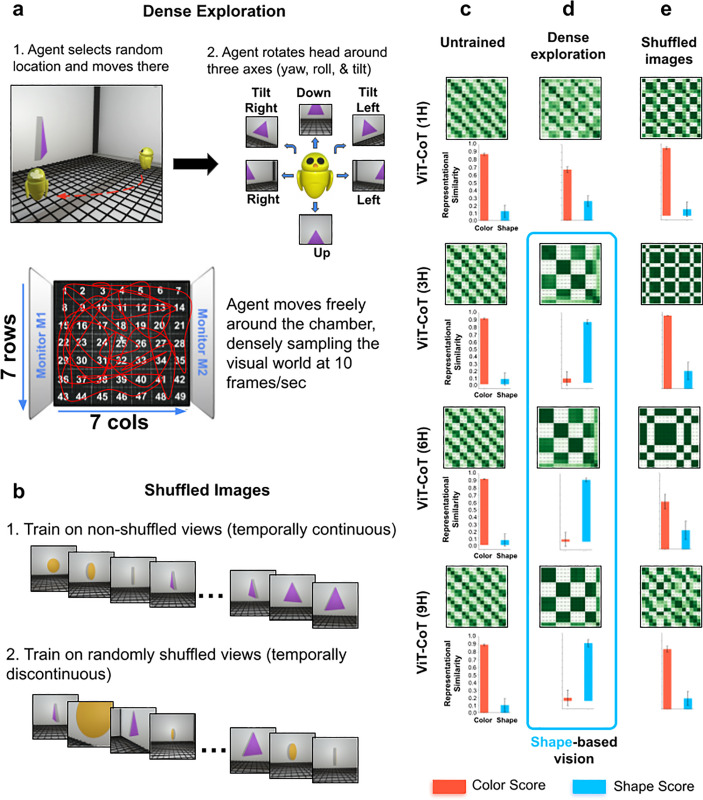
Controlled-rearing experiments on generic fitting models. (a) In the ‘dense exploration’ condition, the agent freely moves around the virtual controlled-rearing chamber, densely sampling the visual experiences available in the chamber with head movements. (b) In the ‘shuffled images’ condition, we randomized the order of the simulated training images to test the importance of temporal continuity on the development of shape-based vision. (c) Untrained generic fitting models had color-based representational spaces, as shown in the RDMs (*top*) and color/shape scores (*bottom*). (d) Generic fitting models trained in the dense exploration condition developed robust shape perception. The one exception was the smallest (1H) model, which failed to develop shape perception. (e) Generic fitting models trained in the shuffled images condition also failed to develop shape perception, highlighting the importance of temporal continuity in shape learning. For all RDMs, the images used to make the RDMs were the same as those used in Fig 1. Error bars denote standard error for each model across the color cells and shape cells shown in Fig 1b, c.

To test this hypothesis, we simulated the controlled rearing study from Wood [11], where chicks developed shape perception after being raised 24/7 in a world with a single object (Fig 3a). We created digital twins (virtual replicas) of the controlled-rearing chambers in a video game engine (Fig 3b), then simulated the visual experiences available in the environment by recording the first-person views acquired by agents moving through the virtual chambers. During the simulation (Fig 4a), the agent densely sampled the visual environment, moving to different random locations in the chamber and moving its head along the three axes of rotation (yaw, pitch, roll). We collected 80K images from the agent while it looked at an animation of a rotating object (the object had two faces, each face with a different color and shape). We then used those images to train generic fitting models (Fig 3c). Finally, we froze the trained models and measured their representational spaces with the object stimuli used to test the chicks (Fig 3d).

During training, the fitting models transformed from color-based (untrained) to shape-based visual systems (Figs 3e and 4d), akin to the models trained on visual experiences from human adults (Fig 1e). This change occurred in all the models except the smallest (1H) architecture size (Fig 4d), consistent with the results from models trained on visual experiences from human adults.

How do generic fitting systems learn so much (shape perception) from so little (visual input of a single object)? One hypothesis is that shape perception requires experience with large numbers of *objects*, but our simulations show that generic fitting models develop shape perception in sparse environments with a single object. A second hypothesis is that shape perception requires experience with large numbers of *environments*, but our simulations show that generic fitting models develop shape perception in a single environment. A third hypothesis is that shape perception requires experience with *natural features*, but the controlled-rearing chambers lacked many natural features, yet the models still learned shape perception. Which experiences, then, transform generic fitting models from color-based to shape-based visual systems?

We hypothesize that the relevant visual experiences for developing shape perception are *diverse temporally linked views*. When embodied agents move, they acquire large numbers of spatially and temporally linked views (retinal images). These views contain information about how object shape changes across viewing situations [22,38–42]. If a model (or brain) could learn from diverse temporally linked views, then they might have all the information they need to develop shape perception.

### Embodied view diversity drives shape learning

To test this embodied view-diversity hypothesis, we trained generic fitting models in altered visual worlds where view diversity was distorted or reduced in four ways. First, to demonstrate that temporal continuity between views—generated naturally by embodiment—is important for learning shape perception, we shuffled the order of the training images, removing the temporal continuity across views (Fig 4b). Shuffling the images prevented learning of shape perception (Fig 4e), indicating that temporal continuity across views drives shape learning in generic fitting systems.

**Fig 5:**
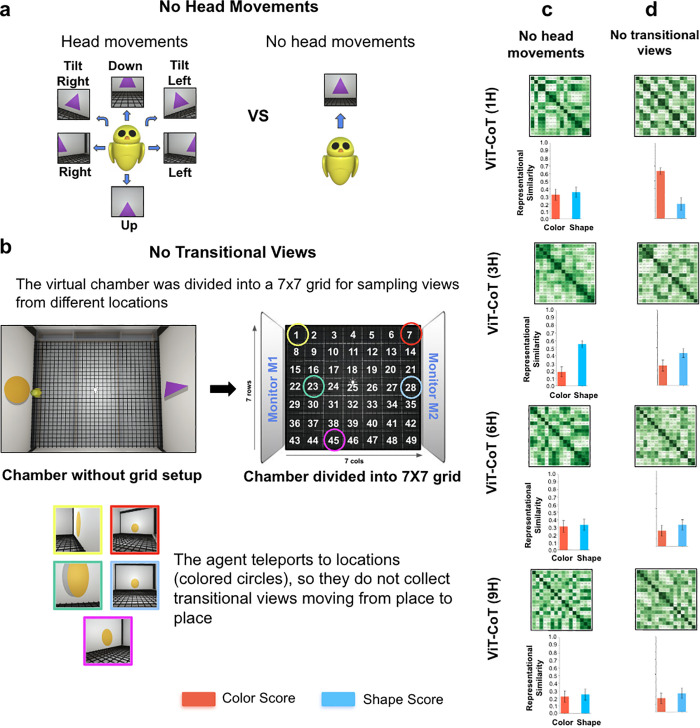
Ablating head movements and transitional views. (a) In the ‘no head movements’ condition, the agent moved freely around the virtual chamber, but rather than performing head movements at each location (as in the dense exploration condition, *left*), the agent stared directly at the object (*right*). (b) In the ‘no transitional views’ condition, the chamber was divided into 49 equally-spaced locations, and the agent teleported to each location. The colored circles and associated images show sample views obtained from five locations. Unlike the dense exploration condition, the agent did not acquire transitional views moving from place to place. (c) Generic fitting models trained in the no head movements condition developed partial sensitivity to shape, as shown in the RDMs (*top*) and color/shape scores (*bottom*). This differs from models trained in the dense exploration condition, which developed robust shape perception (Fig 4d). (d) Generic fitting models trained in the no transitional views condition developed partial sensitivity to shape. Again, this differs from models trained in the dense exploration condition, which developed robust shape perception (Fig 4d). In both conditions, the models partially reduced their weighting of color features (compared to untrained models, Fig 4c). For all RDMs, the images used to make the RDMs were the same as those used in Fig 1. Error bars denote standard error for each model across the color cells and shape cells shown in Fig 1b, c.

Second, we ablated all head movements from the training data, forcing the agents to still their head (camera) while moving around the chamber (Fig 5a). This condition was inspired by the observations of Bambach and colleagues [58], who reported that young children generate diverse head movements when visually inspecting objects. We speculate that this view diversity from head movements provides important image variation for learning object shape. As hypothesized, the models trained on visual experiences lacking head movements failed to develop robust shape perception (although one architecture size did learn partial shape perception, Fig 5c).

Third, we ablated visual transitions moving from one location to the next (Fig 5b). The agent teleported from place to place, without moving smoothly between locations. In the natural world, when agents traverse a path, they see large numbers of diverse, temporally linked views [59]. The view diversity gained moving from place to place might play an important role in learning shape perception. Supporting this hypothesis, when we removed training views generated when moving from place to place, the fitting models failed to develop robust shape perception (Fig 5d).

**Fig 6:**
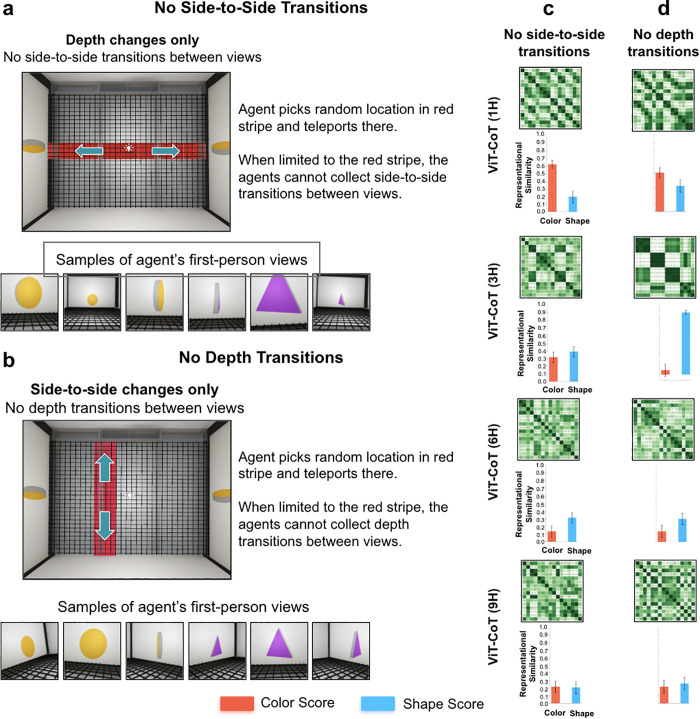
Ablating lateral and depth transitions between views. (a) In the ‘no side-to-side transitions’ condition, the agent teleported to locations within the horizontal red stripe. Since the agent was limited to the red stripe, they could not collect views showing side-to-side transitions of the object. (b) In the ‘no depth transitions’ condition, the agent teleported to locations within the vertical red stripe. Since the agent was limited to the red stripe, they could not collect views showing depth transitions of the object. (c) Generic fitting models trained in the no side-to-side transitions condition developed partial sensitivity to shape, as shown in the RDMs (*top*) and color/shape scores (*bottom*). This differs from models trained in the dense exploration condition, which developed robust shape perception (Fig 4d). (d) Generic fitting models trained in the no depth transitions condition developed partial sensitivity to shape. Again, this differs from models trained in the dense exploration condition, which developed robust shape perception (Fig 4d). The one exception was the 3H model, which developed shape perception. In both conditions, the models partially reduced their weighting of color features (compared to untrained models, Fig 4c). For all RDMs, the images used to make the RDMs were the same as those used in Fig 1. Error bars denote standard error for each model across the color cells and shape cells shown in Fig 1b, c.

Fourth, we reduced view diversity by limiting where the agent could sample views inside the chamber. When we only allowed the agent to teleport to different locations in the depth plane (without side-to-side transitions between views, Fig 6a), the agents failed to develop robust shape perception (Fig 6c). Likewise, when we only allowed the agent to teleport to different locations in the lateral plane (without depth transitions between views, Fig 6b), the fitting models largely failed to learn shape perception (Fig 6d). In the ‘no depth transitions’ condition (Fig 6d), the 3H model developed shape perception, contrary to the 1H, 6H, and 9H models. To test whether this was an anomaly, we trained and tested 10 additional models in this condition. Four of the 10 models developed shape perception, suggesting that shape perception occasionally develops when these models are reared in these conditions. We also trained 10 additional 3H models in the ‘dense exploration’ condition (Fig 4d), where we hypothesized most of the models would succeed. Nine of the 10 models developed shape perception, indicating that shape perception does reliably develop when 3H models are reared in conditions that provide diverse temporarily-linked views.

These controlled-rearing manipulations show that the visual diet available in an impoverished world (a white room with a single object) is sufficient for generic fitting systems to develop shape perception. By adapting to diverse temporally linked views, fitting systems can develop shape perception. As such, when view diversity is ablated or temporal continuity between views is absent, generic fitting models fail to develop shape perception. Our results thus highlight the importance of both the learning machinery (generic fitting mechanisms) and experience (diverse views) in learning shape perception.

To confirm that this pattern generalizes across objects, we reran all the controlled-rearing experiments, but replaced the object in the chamber with a new object (S2a Fig), with different colors and shapes. The experiments replicated the original pattern: untrained fitting models organized objects by color (S2b Fig), whereas trained models organized objects by shape (S2c Fig). The models also largely failed to learn shape perception when their visual diet was altered, including when the views were shuffled (S2d Fig), when transitional views were ablated (S2e Fig), when head movements were ablated (S2f Fig), when side-to-side transitions were ablated (S2g Fig), and when depth transitions were ablated (S2h Fig). Overall, generic fitting models—equipped with hardcoded artificial image augmentations—develop shape perception when their training data contains diverse temporally linked views of the world.

### Small generic fitting models do not learn shape perception

From a fitting perspective, models with more parameters should develop more accurate fits to the environment. The world is a high-dimensional place, so fitting models may need many adjustable parameters to learn accurate high-dimensional representations of the world. While early attempts to model brain development through an evolutionary lens [33,34,60] used small models and tested simple toy behaviors, modern overparameterized machine-learning models (e.g., transformers) can learn to perform a wide range of tasks and perform better at larger scales [61–63]. Overparameterized systems appear important for accurately fitting to the world.

Our results support this view. Unlike the architectures with larger numbers of blocks and attention heads (3 blocks/heads, 6 blocks/heads, 9 blocks/heads), the small architecture (1 block/head) was largely unable to learn shape perception, either from the egocentric visual experiences of human adults (Figs 1e and 2c) or newborn chicks (Figs 4d and S2c). Not all generic fitting systems develop shape perception: their architectures must be sufficiently large (overparameterized) to learn shape-based representational spaces.

This finding supports paradigms in computational neuroscience that use large-scale models to mimic the algorithms in brains [35,64–67]. Brains are over-parameterized, containing billions of neurons (with trillions of adjustable synapses). Thus, large-scale fitting models might be essential for mimicking the core learning machinery in brains. With large-scale fitting models, researchers can directly test fitting theories of development, by running simulations measuring whether generic fitting systems develop brain-like behaviors when ‘reared’ in the same worlds as newborn animals. Since the models are image computable, they produce precise predictions of what generic fitting systems do, and do not, learn from particular experiences.

### Hardcoding spatial priors into fitting models

Generic fitting models (e.g., transformers) reveal what can be learned from experience, when a system starts with no prior domain-specific knowledge. Without hardcoded knowledge, a model must rely on experience alone to provide all the necessary information for learning a mental skill. Importantly, however, there are ways to constrain what generic fitting models learn by using hardcoded priors. For example, as we show below, artificial image augmentations heavily constrain visual development in transformers. Likewise, many researchers in vision science use fitting models with strong hardcoded spatial priors.

To explore the consequences of giving fitting models hardcoded spatial priors, we reran all of the experiments with convolutional neural networks (CNNs), which have hardcoded priors about the spatial structure of natural images, due to their convolutional operation and hierarchical organization [68]. The CNN models had the same temporal learning objective [69] as the transformers, so the CNNs differed from the transformers only in architecture.

**Fig 7:**
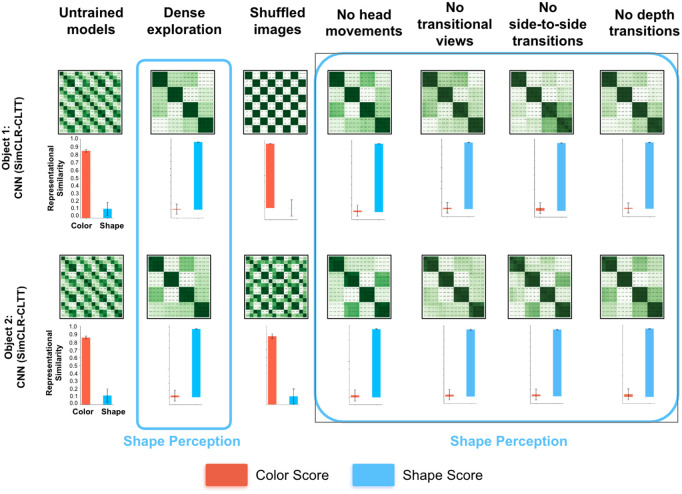
Hardcoding spatial priors into fitting models. Unlike Vision Transformers (ViTs), convolutional neural networks (CNNs) have hardcoded spatial priors. To measure the impact of hardcoding spatial priors into fitting models, we compared ViTs and CNNs across all of the controlled-rearing conditions. The ViTs and CNNs had the same temporal learning objective, so the models differed only in architecture. Unlike the ViTs, which learned shape perception by leveraging view diversity (Figs 4–6), CNNs learned shape perception in nearly all of the controlled-rearing conditions. The one exception was when the training views were shuffled, which prevented shape learning in both CNNs and ViTs. The same pattern emerged with both imprinting objects (*top* and *bottom* rows). Hardcoding spatial priors into fitting models reduces the model’s reliance on view diversity for learning shape perception. For all RDMs, the images used to make the RDMs were the same as those used in Fig 1. Error bars denote standard error for each model across the color cells and shape cells shown in Fig 1b, c.

Unlike the transformers, which learned shape perception by leveraging view diversity, CNNs learned shape perception in nearly all of the controlled-rearing conditions described above, including the conditions with limited view diversity (Fig 7). The one exception was when the training views were shuffled, which prevented shape learning in both CNNs and transformers. This pattern emerged when the CNNs were trained on either of the imprinting objects. Thus, hardcoding spatial priors into fitting models can reduce the model’s need for view diversity during learning.

Are brains more like CNNs (hardcoded spatial priors) or transformers (generic fitters)? There is growing evidence that brains are transformer-like in many ways: for example, transformers learn representations similar to those found in the hippocampus, including grid, place, border, and direction cells [70]; transformer computations mimic the neuron-astrocyte network in brains [71] and implement common context encoding as cortical waves [72]; and transformers can learn core visual skills in the same impoverished environments as newborn animals [73].

There is also evidence that newborn animals leverage diverse temporally linked views to learn how to see [74–76][77–78], akin to transformers. Indeed, recent studies show that ViTs perform as well (or better) than CNNs on visual recognition tasks [79–81] and rival CNNs in predicting neural activation patterns in brains [82]. Digital twin studies provide converging evidence favoring ViTs over CNNs: when reared and tested in the same visual environments as newborn chicks, ViTs—but not CNNs—mirror the chicks’ successes and failures on object recognition tasks [83].

Importantly, ViTs *develop* CNN-like retinotopic and hierarchical architectures when trained on egocentric visual experiences [84]. Thus, CNN-like architectures are statistical products of more generic (transformer-like) fitting systems adapting to experience. We thus speculate that the core learning algorithm in brains is transformer-like, and this core produces a CNN-like architecture during prenatal development.

### Temporal learning drives shape perception

Was temporal learning important for the fitting models? To test this, we evaluated whether non-temporal learning models develop shape perception in these impoverished visual worlds. When given the same training data as the temporal learning models, we found that CNN-based autoencoders (S3a Fig) develop partial shape perception, and GreedyInfoMax (S3b Fig) fails to develop shape perception. After training, the models’ representational spaces were largely color-based (S3c Fig). These models were trained with the same artificial data augmentations used above, but still failed to develop shape perception. While non-temporal models can eventually learn robust shape representations from extensive training data [85], our results suggest that temporal learning spurs development of shape perception in the sparse environments faced by newborn animals.

### Measuring the impact of hardcoded artificial image augmentations on shape perception

**Fig 8:**
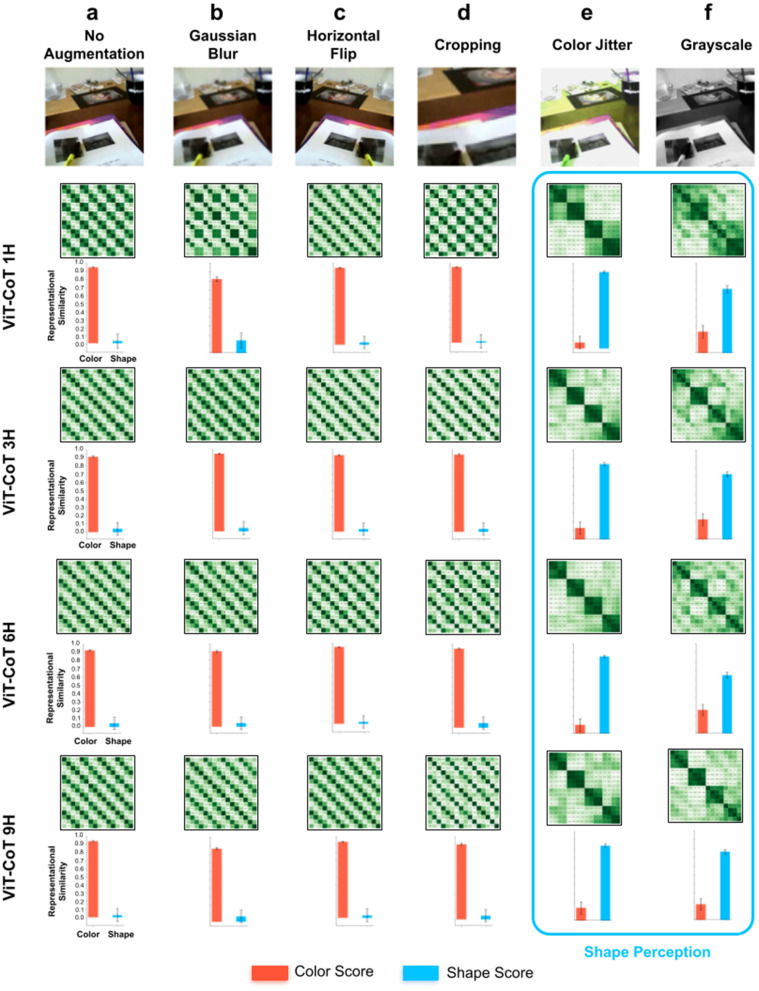
Training generic fitting models with different artificial image augmentations. (a) Generic fitting models trained with no artificial image augmentations developed color-based representational spaces, as shown in the RDMs (*top*) and color/shape scores (*bottom*). (b-d) Likewise, generic fitting models trained with gaussian blur, horizontal flip, or random cropping developed color-based representational spaces. (e-f) Conversely, generic fitting models trained with color jitter or grayscale developed shape-based representational spaces. For all RDMs, the images used to make the RDMs were the same as those used in Fig 1. Error bars denote standard error for each model across the color cells and shape cells shown in Fig 1b, c.

As discussed above, modern computer vision models artificially increase view diversity by adding hardcoded image augmentations during training [48–50]. To test how these different sources of view diversity impact learning of shape perception, we measured the contribution of each image augmentation separately (Fig 8). For each model, we used one image augmentation while training on the egocentric visual experiences of human adults. We also trained baseline models without any artificial image augmentations. Generic fitting models trained with no artificial image augmentations did not learn shape perception: the models’ representational spaces were color-based (Fig 8a). Likewise, three of the image augmentations (gaussian blur, random resized crop, random horizontal flip) had no benefit on learning shape perception (Fig 8b–d). Two image augmentations—grayscale and color jitter—led to substantial improvements in shape learning (Fig 8e and 8f).

To check that this pattern generalizes across datasets (different visual experiences), we trained models on two new video datasets collected from human adults wearing head-mounted cameras. The models showed the same pattern in both datasets (S4 and S5 Figs), developing shape perception only when views were augmented with grayscale or color jitter. Thus, some kinds of view diversity matter, and others do not, for learning shape perception.

### Replacing artificial image augmentations with artificial retinas

The finding that grayscale and color jitter permit models to develop shape perception is intriguing because human retinas contain ~60 million rods and ~3 million cones (~95% rods, ~ 5% cones) [77]. Cone density peaks at the fovea (~150,000–180,000 cones/mm² at the center) and falls with eccentricity to ~6,000 cones/mm² at 1.5 mm and ~2,500 cones/mm² near the ora serrata. In contrast, rod density is highest in a ring 3–5 mm from the fovea (~150,000 rods/mm²) and declines to ~30,000–40,000 rods/mm² toward the periphery. While cones are concentrated centrally, they are present across the retina; color sensitivity is maximal near fixation and diminishes with eccentricity. This spatially varying mix of rods and cones naturally produces grayscale-versus-color contrasts akin to the augmentations that help transformers learn shape.

**Fig 9:**
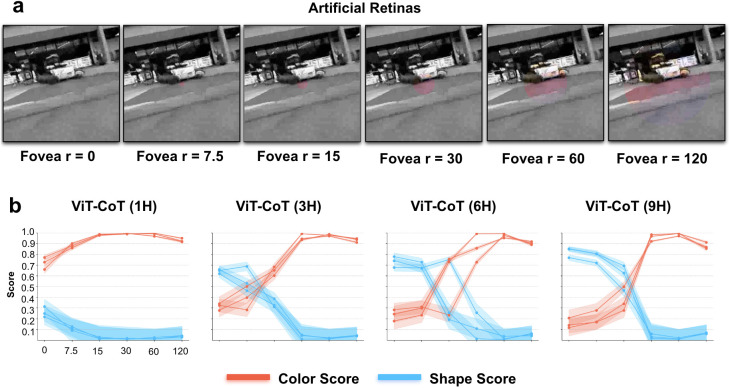
Filtering raw visual experiences through artificial retinas. (a) We created six artificial retinas, each with a different-sized foveal region. The foveal region had color filters, mimicking the high density of cone (color) receptors in the retina. The periphery had grayscale filters, mimicking the high density of rod cells in the retina. The eye filter with a radius of 7.5 units is the closest match to human eyes. (b) Color and shape sensitivity of generic fitting models trained on human visual experiences filtered through artificial retinas. All models, except the smallest model (1H), develop shape perception when trained using artificial retinas with small foveal regions (akin to human eyes). Models trained using artificial retinas with larger foveal regions failed to develop shape perception. Error bars denote standard error for each model across the color cells and shape cells shown in Fig 1b, c.

To test this possibility, we first built a toy artificial retina that roughly mimicked the arrangement of cones on the human eye (Fig 9a). We also built unnatural artificial retinas with larger color regions than are seen in human eyes. We filtered the egocentric visual experiences from human adults through the artificial retinas. This produced visual inputs that either roughly matched (human artificial retina) or did not match (unnatural artificial retinas) the visual inputs received by visual systems, after light rays are filtered through the retina. We removed all other forms of image augmentation; the only image augmentations available to the model were those performed by the artificial retinas (which were applied on 100% of the training images).

Fig 9b shows the representational spaces learned by the same models equipped with different artificial retinas (all models were tested with the same images used to test all other models, with no artificial retina applied). Only the models that learned with artificial retinas that roughly matched human eyes—with cones (color vision) covering just 1–2° of the visual field—developed shape perception. Models with larger color regions learned color-based representational spaces. These findings accord with studies showing that color vision can cause both brains and models to develop overly strong reliance on chromatic cues when learning object recognition, hindering the development of core visual skills [86].

The architecture of retinal circuits is relatively fixed [87], so there is little plasticity in the retina compared to the cortex. Retinas can thus be subject to precise evolutionary manipulation, allowing natural selection to shape eyes to produce filtering effects that enhance survival. Researchers have long appreciated the importance of eyes for creating clear images, detecting motion, depth, and color, and for adapting to changing light conditions [88]. We show that retinas also have a direct causal role in developing shape perception.

Our retina-manipulation experiments demonstrate that eyes can heavily determine whether a latent representational space is color-based versus shape-based. The model architectures, objective functions, learning rules, and embodied training data were identical except for their artificial retinas; the retina filters alone determined the nature of the learned representational space. Given that artificial retinas are almost never used in computer vision (but see ref [89,90]), we argue for increased attention to visual sensors that transform raw visual experience in biologically plausible ways.

### Optimizing view diversity with biologically plausible artificial retinas

**Fig 10:**
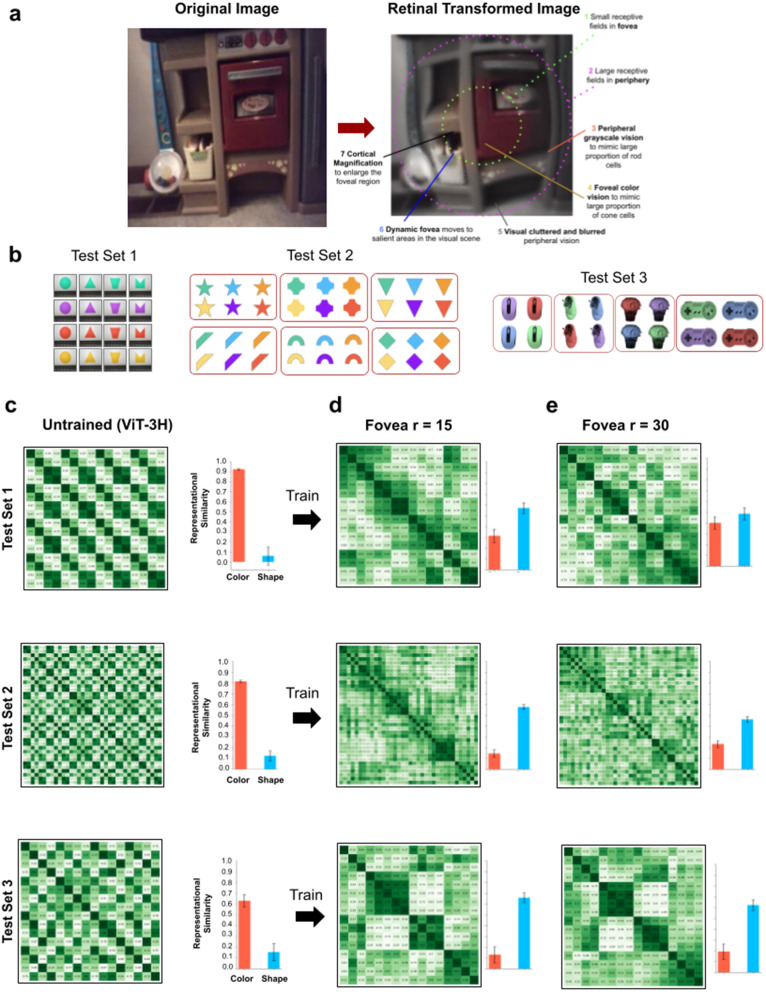
Realistic artificial retinas. (a) Realistic artificial retinas convert RGB images into retinal-formatted images, akin to the transformations performed by biological retinas. For this experiment, we used fovea sizes of 15 and 30, 99% of cones in fovea and 1% of cones in periphery, 1% of rods in fovea and 99% of rods in periphery, larger receptive fields in the periphery versus fovea, visual crowding in the periphery, and a dynamic fovea that moved to salient regions across successive images. (b) We tested the untrained and trained models across the three stimuli sets. (c) Untrained models were color-based, whereas trained models (d-e) developed forms of shape perception. Error bars denote standard error for each model across the color cells and shape cells shown in Fig 1b, c.

To tackle this challenge, we built a more biologically plausible artificial retina (Fig 10a). The toy retinas used above were crude approximations of biological retinas. From a shape learning perspective, real retinas have many features that impact how input is processed [91]. First, receptive field sizes vary across the retina: in the fovea, receptive fields are small and support high-acuity vision, whereas in the periphery, receptive fields become progressively larger—ranging from approximately 1–5° of visual angle and extending up to 10° or more in the far periphery. This non-uniform sampling changes the granularity of spatial information across the visual field. Second, the distribution of photoreceptors shapes visual input, particularly with respect to color and motion processing. While cones—responsible for high-acuity color vision—are densely packed in the fovea, they constitute only ~5% of photoreceptors across the entire retina. In contrast, rods, which are sensitive to luminance but not color, make up roughly 95% of photoreceptors and dominate the periphery. Third, the fovea occupies a small portion of the visual field (1–2° of visual angle), meaning that detailed, color-sensitive vision is largely restricted to a narrow region. Fourth, the visual system samples the world through eye movements (saccades), actively shifting the fovea to bring regions of interest into high-resolution focus. Fifth, biological retinas have cortical magnification and retinotopic warping. Because retinal ganglion cells are distributed with much higher density in central vision than in the periphery [92], equal areas of the retina project to unequal cortical areas—a phenomenon known as cortical magnification [93]. In effect, the cortical “image” is a warped version of the retinal image, with central regions expanded and peripheral regions compressed. As the eyes move across a scene, this cortical image shifts dynamically with gaze, magnifying regions around the fixation point [94]. We incorporate this principle by performing image transformations into cortical coordinates, creating a biologically inspired warping that reflects the natural retinotopic organization of vision.

We evaluated the impact of this realistic artificial retina on the three stimuli sets used above (Fig 10b). Fig 10c shows the representational spaces of untrained models (all models were color-based). Fig 10d shows the representational spaces of models trained with the artificial retinas. Models with both smaller (r = 15, Fig 10d) and larger (r = 30, Fig 10e) foveal radii developed shape perception.

Intriguingly, the toy artificial retinas with r = 30 foveal radii developed color-based representational spaces (Fig 9), whereas the more realistic artificial retinas with r = 30 foveal radii developed shape-based representational spaces (Fig 10e). This pattern suggests that making artificial retinas more realistic can lead to systematic improvements in shape learning. Future studies could independently manipulate different retinal features to further pinpoint the particular retinal features that drive shape learning.

## Discussion

We used image-computable generic fitting models to simulate the origins of shape perception. We found that shape perception develops when generic fitting systems adapt to embodied visual experiences, including visual experiences from human adults and newborn chicks. We systematically removed different kinds of experience from the training data and found that experience with diverse temporally linked views drives shape learning. We also showed that human eyes produce the type of view diversity needed to learn shape perception. Thus, shape perception develops from three ingredients: (1) generic fitting systems, (2) embodied visual experiences, and (3) biologically plausible sensors. We provide a conceptually simple—yet image computable—account of the origins of shape perception.

Our work builds on recent advances in machine learning, where fitting models have achieved impressive performance on object recognition tasks [95–99], including tasks focused on shape recognition [85,100]. These models are typically trained on large, uncontrolled datasets, paired with hardcoded artificial image augmentations, that are unrepresentative of the visual experiences and internal data augmentations of humans and animals. Our study confirms that machine learning models can still learn effectively when trained on biologically plausible data streams, by leveraging body sensors that augment sensory input at the front end of the visual system.

Our results show how controlled rearing can be used to probe the precise role of experience in learning for both brains and models. Theorists have long recognized that visual experience is rich, but it was not previously possible to characterize which particular experiences drive learning of core visual skills. Nor was it possible to determine whether newborns have access to sufficiently rich experiences for learning shape perception, leading to centuries of debate about whether brains need innate knowledge for learning to perceive and understand the world. By raising animals and generic fitting models in the same environments, we provide direct evidence that shape perception is readily learnable from the visual experiences of newborn animals, in the absence of innate shape primitives. Generic fitting systems leverage diverse temporally linked views to learn shape-based representational spaces.

Linking developmental psychology to machine learning opens exciting paths for probing the core learning machinery in brains. Researchers can raise models in the same environments as animals, then measure whether the animals and models learn the same mental skills. Models can also be reared in environments that are not possible (e.g., teleporting agents from place to place), giving scientists new tools for studying the origins of knowledge.

Our results also suggest that bodies will be important for reverse-engineering intelligence [89]. Researchers have largely focused on architectures, objective functions, and learning rules as the relevant dimensions for building task-performing models of visual systems [66]. We show that both visual diet and sensors also have major impacts on the representational spaces learned by models, even when architecture, objective function, and learning rules are identical across models. Sensors transform raw visual signals, and this transformed input (not the raw visual signal) is sent to the brain. Likewise, bodies determine and constrain the flow of visual experiences acquired by the brain. We speculate that reverse engineering vision will require filtering raw visual input through biologically accurate sensors, equipped to animal-like morphologies. These artificial bodies should transform input for models the same way that eyes and bodies transform input for brains.

Ultimately, our simulations show that shape perception can be the product of generic fitting. The fitting process of brains (*variation* over synaptic connections combined with environmentally driven *selection* of connections) is comparable to the fitting process in evolution (*variation* over genes and environmentally driven *selection* of genes). Thus, development and evolution can be united under a common fitting framework, with shared general principles [35–37]. Under this view, knowledge develops as the brain gradually fits to the organism’s prenatal and postnatal world. Researchers have observed significant changes in shape perception across the first 4–5 years of human development [26]. Future studies could explore whether generic fitting models produce the same developmental trajectories of shape perception as humans, gradually developing more complex shape perception as their representational spaces achieve better fits to the environment.

### Limitations and conclusions

Our study provides an image-computable origin story for shape perception, opening several directions for future research. First, there are many ways to compare learning and representational structures across biological and artificial systems [101,78]. For example, one technique is to compare representational axes across biological and artificial systems [101]. Another approach is to use formal benchmarks from human adults to compare shape perception skills across brains and machines [85]. Since our goal was to explore the origins of shape perception, we focused on the initial ingredients that permit newborn-like shape perception to develop in artificial neural networks. Thus, future work is needed to test whether our results generalize across this broader range of metrics, stimuli sets, and age ranges.

Second, additional research is needed to close the “body gap” between biological and artificial systems. Our biologically inspired retinas captured many—but not all—properties of biological retinas. For instance, they did not include event-based processing [102] or capture saturation effects, where photoreceptors vary their activation across lighting conditions (e.g., rods dominate low-light vision but saturate during daylight). Nor did our artificial retinas undergo developmental change. In humans, vision gradually increases in acuity, color sensitivity, and contrast sensitivity [103]. When CNNs are trained on visual diets that mimic this developmental progression, the models develop robust shape perception, including human-like shape biases, resistance to image corruptions, and resilience to adversarial attacks [49,86,104]. Early receptor limitations (e.g., blur, reduced contrast, and restricted color) guide models toward shape-based representations, rather than brittle, texture-based shortcuts. Future work could incorporate these developmental changes into artificial retinas.

Third, while our results show that generic fitting systems, embodied visual experiences, and biologically plausible sensors are *sufficient* to develop shape perception, they do not show that these ingredients are *necessary*. Indeed, most computer vision models achieve strong performance when trained on static image datasets and artificial image augmentations. But this is not how humans learn. Humans develop vision through continuous streams of egocentric input, in the absence of hardcoded image augmentations. Our goal was not to model how vision can be optimized *in silico*, but rather to test whether human-like shape perception can develop under the realistic constraints of embodied visual input and biologically inspired sensors.

In sum, we identify learning machinery, experiences, and sensors that are sufficient to develop shape perception. By showing that shape perception can develop from realistic streams of sensory input filtered through biologically plausible sensors, our work bridges debates about innateness and learning, and offers a blueprint for building artificial agents with human-like shape perception. Our results also support a broader vision: development and evolution can be understood as parallel fitting processes governed by the same general principles, yielding a simple yet powerful account of how minds learn to perceive and understand the world.

## Methods

### Egocentric human videos

We used the UT Ego dataset, which consists of videos collected using head-mounted cameras. The recordings capture the daily activities of graduate students. Each video was ~ 2 hours and 15 minutes long (135 minutes) and included various environments such as study rooms, outdoor scenes, and lecture halls. To train the models with UT Ego data, we converted the videos into image sequences at 64 × 64 resolution (10 frames/sec), then selected the first 80,000 images from each video for the training set. These images were sequentially passed into the models for training.

### Representation Dissimilarity Matrix (RDM)

To create Representation Dissimilarity Matrices (RDMs), we first designed a 16 × 16 matrix, with each row and column showing the 16 test objects (4 shapes × 4 colors). We then passed the test images through the frozen encoder and extracted the feature embeddings from the last layer of the model. To normalize the features, the embeddings from all the test images were concatenated into a single vector, and we calculated the mean of this concatenated vector and subtracted it from each individual image vector, centering the values around zero. Finally, we calculated the pairwise cosine similarity between each pair of test images to plot the RDMs. The cells within the RDMs are color-coded based on the strength of the cosine similarity.

The bar graphs were created using the values from the RDM cells. We used bar graphs to show the model’s color score (average similarity between objects that matched in color, Fig 1b) and shape score (average similarity between objects that matched in shape, Fig 1c).

### Controlled-rearing chambers

*Animal Chamber.* For details on the chick study, see Wood [11]. The chicks were raised in automated controlled-rearing chambers measuring 66 cm (length) × 42 cm (width) × 69 cm (height). The chambers were made from white, high-density plastic and did not contain any real-world (solid and bounded) objects. To present object stimuli to the chicks, Wood [11] projected virtual objects on two 19’‘ display walls (LCD monitors, 1440 × 900 resolution) on opposite sides of the chamber. Food and water were provided *ad libitum* in transparent troughs in the floor. Grain was chosen as the food because it does not maintain a rigid, bounded shape and thus does not behave like a solid object. The chamber floors were made of black wire mesh supported by transparent beams. Micro-cameras installed in the ceilings were used to record the chicks’ behavior 24/7, using image-based tracking software.

During the input phase, each chick’s visual object experience was limited to a single virtual object with two faces. The object rotated continuously, revealing a different color and shape on each face. The object appeared on one wall at a time, switching walls every 2 hours. The object rotated around a frontoparallel vertical axis and completed a full rotation every 6 seconds. The edge of the object shown during rotations from one face to the other looked identical. Because the object had two faces, it changed smoothly from one 2D shape-color combination to another. On average, the object measured 9 cm (length) × 7 cm (height). It was displayed on a uniform white background.

*Virtual Chamber.* We designed a digital twin (virtual replica) of the animal chambers using the Unity Game engine (Fig 3). Like the real-world chambers, the virtual chamber had four walls: two display walls and two white walls. The display walls were virtual monitors that projected objects to the virtual chick agent, which collected first-person images as it moved around the chamber. The floor of the chamber was textured with a black wire mesh surface, and one side of the chamber had food and water troughs that matched the real-world chambers.

The virtual chamber had two cameras for recording the agent’s behavior. The first camera was placed in the ceiling to capture a top-down view of the agent as it moved around the chamber. The second camera was placed in the agent’s head (facing forward) to collect first-person views as the agent explored the chamber. We collected 80,0000 first-person (RGB) training images for each of the rearing conditions described below. For the t-SNE visualizations, we used 29 test images per object. For the RDM analyses, we used a single test image per object (Fig 1b).

### In silico controlled-rearing conditions

*Dense exploration.* In the dense exploration condition, the agent systematically explored the chamber (Fig 4a and 4d). During the simulation, the agent picked a random location inside the chamber and moved to that location at a speed of 1.5 units/second. While moving, the agent kept its gaze fixed on the object shown on the monitor. After reaching the selected location, the agent rotated its head along all three axes (yaw, roll, and tilt). These head movements lasted for 9.5 seconds. We sampled the visual world at 10 images/second.

This dense exploration condition canvassed the range of visual experiences of the object that chicks could acquire in the chamber. The approach did not directly simulate a specific chick’s visual experiences. The approach also did not capture views chicks may have seen of their own bodies (e.g., wings, feet). Our virtual agent could not see its body, so its visual diet was limited to views of the object and chamber. As such, this condition established a baseline of what could be learned when a model has access to the same visual environment as newborn chicks.

*Shuffled views.* We took the images from the dense exploration condition and shuffled the images within the batches after each training epoch (Fig 4b and 4e). This removed the temporal continuity between the images.

*No head movements.* This condition was similar to the dense exploration condition, but the agent stared at the object (rather than moving its head) for 9.5s after reaching each randomly selected location (Fig 5a and 5c).

*No transitional views.* We divided the virtual chamber into 49 equally spaced positions (7 × 7 grid). During the simulation, the agent randomly chose a position, but instead of gradually moving to the location (as in the dense exploration condition), the agent teleported directly to the location (Fig 5b and 5d). Thus, the agent did not obtain views moving from place to place. The agent did perform head movements at each location.

*No views of side-to-side transitions.* This condition further restricted the diversity of views obtained by the agent, by preventing the agent from moving from side to side in the chamber (Fig 6a and 6c). Within the 7 × 7 grid described above, the agent could only teleport to tiles in the middle row of the chamber grid. Thus, the agent could move closer and farther away from the object, by randomly selecting and teleporting to the middle tiles.

*No views of depth transitions.* This condition mirrored the condition above, but instead of removing side-to-side transitions, we removed transitions in the depth plane (Fig 6b and 6d). Within the 7 × 7 grid described above, the agent could only teleport to tiles in the middle column of the chamber grid. The agent moved to the left and right of the object, by randomly selecting and teleporting to lateral tiles.

### Models

*Vision Transformer (ViT-CoT).* We systematically varied the number of attention heads and transformer layers to create different ViT architecture sizes. For instance, ViT-CoT (1H) had one attention head and one transformer layer; ViT-CoT (3H) had three attention heads and three transformer layers; and ViT-CoT (9H) had nine attention heads and nine transformer layers. The ViT-CoTs were trained using a self-supervised contrastive learning through time objective function. The frames in the dataset were not shuffled, thereby preserving the temporal structure of the embodied data streams. To avoid hardcoding spatial bias in ViT-CoTs, no convolutional layers were used to generate image patches. The models were trained on 64 × 64 resolution images with a patch size of 8 × 8. All models were trained using the Adam optimizer with a constant learning rate of 0.0001. We report hyperparameters used to train ViTs in Table 1.

**Table 1:** Hyperparameter details of the models.

Model	Params (M)	Layers	Attention Heads	Batch Size
ViT-CoT (1H)	5.8	1	1	128
ViT-CoT (3H)	16.9	3	3	128
ViT-CoT (6H)	36.4	6	6	128
ViT-CoT (9H)	59.4	9	9	128
SimCLR-CLTT	7.9	10	NA	512
AE	15.5	10	NA	128
GIM	1.7	10	NA	32

*Convolutional Neural Network (SimCLR-CLTT).* We created a custom CNN architecture (ResNet-10), which consists of two residual blocks inspired by the default ResNet-18 architecture. Similar bridge connections between the residual blocks were implemented as in default ResNets. Like the ViT-CoTs, the SimCLR model was trained using contrastive learning through time. To train SimCLR-CLTT, we used a learning rate scheduler with a warm-up period of 5 epochs. We report hyperparameters of SimCLR-CLTT in Table 1.

*GreedyInfoMax (GIM).* The GIM model consists of gradient-isolated CNN modules, each optimized with an individual contrastive loss objective. A gradient blocker prevents the backward flow of gradients, stopping backpropagation between modules. We trained a 10-layer GIM model with a batch size of 32. No temporal learning was used in training.

*Convolutional Autoencoder (AE).* We designed a custom CNN architecture, ResNet-10, where the encoder of the autoencoder had 10 trainable layers. After training, we removed the decoder and tested only the embeddings from the encoder. No temporal learning was used in training, similar to GIM.

### Training details

Each model was trained with three different seeds for 100 epochs using a single NVIDIA A10 GPU. The number of trainable parameters for each model is reported in Table 1. We used data augmentation methods from the torchvision library for training the models. All the models were trained using a temporal window size of 3 images and an Adam optimizer. For ViT-CoTs, we used a constant learning rate of 0.0001, whereas for SimCLR-CLTT we used a learning rate scheduler.

### Artificial retinas

The artificial retinas were designed with separate processing streams for the foveal and peripheral regions. In the periphery, input images were converted to grayscale to mimic rod cell processing, while the fovea preserved full color to simulate cone cell function. To define these regions, we first generated a coordinate grid for every image pixel. Using a predefined fovea center and radius, we calculated the squared distance of each pixel from the center. Pixels within the foveal radius were assigned to a binary fovea mask, with the remaining pixels forming a complementary peripheral mask. Six artificial retinas were constructed by systematically varying the foveal radius (0, 7.5, 15, 30, 60, and 120 pixels). Each filter was applied to 320 × 320 images, which were subsequently downsampled to 64 × 64 resolution for model training.

To build biologically inspired retinas, we incorporated several hallmark features of the human eye. These included a clear division between foveal and peripheral regions, with the fovea receiving higher resolution; cortical magnification to emphasize the central visual field; visual clutter and Gaussian blur applied selectively to the periphery; dynamic foveation driven by optic flow across consecutive frames; distinct processing of cone- and rod-like inputs in their respective regions; and a smooth, continuous transition between foveal and peripheral zones.

For the dynamic foveation functionality, we used the RAFT model [105] to compute the optical flow between two consecutive frames (*frame*_*t*_*, frame*_*t+*1_), and generate a saliency map. We then used the saliency map to identify the pixels with the highest flow magnitude, corresponding to the most prominent movement in the scene. Finally, we dynamically positioned the fovea at this location. The visual clutter effect was inspired by [106], and in our implementation, we applied a radial distortion to each pixel in the peripheral region, with the distortion intensity adjusted by the user. For the cortical magnification effect, we applied a falloff function exclusively to pixels in the foveal region. The falloff function calculates the distance of each pixel from the fovea center and gradually expands the pixel closer to the center, creating a zoomed-in effect for high-resolution field of vision.

All the features of the artificial retina were developed using functions from pytorch and torchvision libraries, operating over a single NVIDIA A10 GPU device.

### Statistical tests

The color scores, shape scores, and statistical analyses for all experiments are reported in Tables 2 and 3.

**Table 2:** Results of paired *t*-tests comparing between shape and color scores within each model architecture and training condition.

						Paired *t*-test Statistics		
Figure	Model Architecture	Training Condition	Mean Shape Score	Mean Color Score	Mean Difference (Shape-Color)	*t*	*p*	*df*
**Fig 1**	**Trained ViT 1H**		0.14	0.81	-0.68	-22.84	0.00	2
**Fig 1**	**Trained ViT 3H**		0.65	0.23	0.42	20.18	0.00	2
**Fig 1**	**Trained ViT 6H**		0.71	0.21	0.50	7.14	0.02	2
**Fig 1**	**Trained ViT 9H**		0.63	0.30	0.33	5.18	0.04	2
**Fig 1**	**Untrained ViT 1H**		0.08	0.91	-0.83	-44.54	0.00	2
**Fig 1**	**Untrained ViT 3H**		0.07	0.90	-0.83	-39.87	0.00	2
**Fig 1**	**Untrained ViT 6H**		0.07	0.90	-0.83	-250.00	0.00	2
**Fig 1**	**Untrained ViT 9H**		0.07	0.91	-0.85	-127.00	0.00	2
**Fig 2**	**Trained ViT 1H**		0.28	0.47	-0.19	-6.08	0.03	2
**Fig 2**	**Trained ViT 3H**		0.69	0.16	0.54	18.47	0.00	2
**Fig 2**	**Trained ViT 6H**		0.70	0.15	0.55	14.22	0.00	2
**Fig 2**	**Trained ViT 9H**		0.66	0.17	0.49	15.51	0.00	2
**Fig 2**	**Untrained ViT 1H**		0.12	0.68	-0.55	-62.74	0.00	2
**Fig 2**	**Untrained ViT 3H**		0.14	0.63	-0.49	-84.87	0.00	2
**Fig 2**	**Untrained ViT 6H**		0.13	0.66	-0.52	-59.34	0.00	2
**Fig 2**	**Untrained ViT 9H**		0.14	0.64	-0.51	-57.45	0.00	2
**Fig 3**	**Trained ViT 6H**		0.89	0.04	0.86	46.16	0.00	2
**Fig 3**	**Untrained ViT 6H**		0.07	0.90	-0.83	-250.00	0.00	2
**Figs 4–6**	**Trained ViT 1H**	**Dense Exploration**	0.26	0.66	-0.40	-40.00	0.00	2
**Figs 4–6**	**Trained ViT 1H**	**No Depth Transitions**	0.41	0.44	-0.04	-0.30	0.80	2
**Figs 4–6**	**Trained ViT 1H**	**No Head Movements**	0.32	0.39	-0.07	-1.39	0.30	2
**Figs 4–6**	**Trained ViT 1H**	**No Side-to-Side Transitions**	0.25	0.54	-0.29	-1.86	0.20	2
**Figs 4–6**	**Trained ViT 1H**	**No Transitional Views**	0.26	0.56	-0.30	-2.49	0.13	2
**Figs 4–6**	**Trained ViT 1H**	**Shuffled Images**	0.09	0.93	-0.84	-84.00	0.00	2
**Figs 4–6**	**Trained ViT 3H**	**Dense Exploration**	0.62	0.12	0.50	3.28	0.08	2
**Figs 4–6**	**Trained ViT 3H**	**No Depth Transitions**	0.86	0.06	0.80	40.00	0.00	2
**Figs 4–6**	**Trained ViT 3H**	**No Head Movements**	0.55	0.18	0.37	3.77	0.06	2
**Figs 4–6**	**Trained ViT 3H**	**No Side-to-Side Transitions**	0.46	0.23	0.23	2.17	0.16	2
**Figs 4–6**	**Trained ViT 3H**	**No Transitional Views**	0.44	0.22	0.23	3.39	0.08	2
**Figs 4–6**	**Trained ViT 3H**	**Shuffled Images**	0.18	0.85	-0.67	-17.13	0.00	2
**Figs 4–6**	**Trained ViT 6H**	**Dense Exploration**	0.84	0.06	0.78	10.31	0.01	2
**Figs 4–6**	**Trained ViT 6H**	**No Depth Transitions**	0.24	0.23	0.01	0.13	0.91	2
**Figs 4–6**	**Trained ViT 6H**	**No Head Movements**	0.27	0.23	0.04	2.20	0.16	2
**Figs 4–6**	**Trained ViT 6H**	**No Side-to-Side Transitions**	0.23	0.19	0.04	0.41	0.72	2
**Figs 4–6**	**Trained ViT 6H**	**No Transitional Views**	0.27	0.23	0.03	1.05	0.40	2
**Figs 4–6**	**Trained ViT 6H**	**Shuffled Images**	0.36	0.48	-0.12	-0.44	0.71	2
**Figs 4–6**	**Trained ViT 9H**	**Dense Exploration**	0.82	0.03	0.78	58.75	0.00	2
**Figs 4–6**	**Trained ViT 9H**	**No Depth Transitions**	0.20	0.22	-0.03	-0.60	0.61	2
**Figs 4–6**	**Trained ViT 9H**	**No Head Movements**	0.19	0.21	-0.02	-0.69	0.56	2
**Figs 4–6**	**Trained ViT 9H**	**No Side-to-Side Transitions**	0.20	0.25	-0.05	-1.89	0.20	2
**Figs 4–6**	**Trained ViT 9H**	**No Transitional Views**	0.18	0.23	-0.05	-0.73	0.54	2
**Figs 4–6**	**Trained ViT 9H**	**Shuffled Images**	0.21	0.60	-0.39	-2.24	0.15	2
**Figs 4–6**	**Untrained ViT 1H**		0.08	0.91	-0.83	-44.54	0.00	2
**Figs 4–6**	**Untrained ViT 3H**		0.07	0.90	-0.83	-39.87	0.00	2
**Figs 4–6**	**Untrained ViT 6H**		0.07	0.90	-0.83	-250.00	0.00	2
**Figs 4–6**	**Untrained ViT 9H**		0.07	0.91	-0.85	-127.00	0.00	2
**Fig 7**	**Trained SimCLR-CLTT 10L**	**Dense Exploration**	0.97	0.03	0.94	86.92	0.00	5
**Fig 7**	**Trained SimCLR-CLTT 10L**	**No Depth Transitions**	0.97	0.03	0.94	83.52	0.00	5
**Fig 7**	**Trained SimCLR-CLTT 10L**	**No Head Movements**	0.96	0.03	0.94	222.15	0.00	5
**Fig 7**	**Trained SimCLR-CLTT 10L**	**No Side-to-Side Transitions**	0.94	0.03	0.91	184.73	0.00	5
**Fig 7**	**Trained SimCLR-CLTT 10L**	**No Transitional Views**	0.96	0.02	0.94	111.08	0.00	5
**Fig 7**	**Trained SimCLR-CLTT 10L**	**Shuffled Images**	0.14	0.84	-0.70	-9.67	0.00	5
**Fig 7**	**Untrained SimCLR-CLTT 10L**		0.18	0.78	-0.60	-6.88	0.02	2
**Fig 8**	**Trained ViT 1H**	**Color Jitter**	0.84	0.15	0.69	18.04	0.00	2
**Fig 8**	**Trained ViT 1H**	**Cropping**	0.01	0.98	-0.96	-144.50	0.00	2
**Fig 8**	**Trained ViT 1H**	**Gaussian Blur**	0.03	0.94	-0.92	-103.94	0.00	2
**Fig 8**	**Trained ViT 1H**	**Grayscale**	0.62	0.34	0.28	9.33	0.01	2
**Fig 8**	**Trained ViT 1H**	**Horizontal Flip**	0.06	0.93	-0.87	-150.69	0.00	2
**Fig 8**	**Trained ViT 1H**	**No Augmentation**	0.03	0.94	-0.92	-103.94	0.00	2
**Fig 8**	**Trained ViT 3H**	**Color Jitter**	0.78	0.18	0.60	8.14	0.01	2
**Fig 8**	**Trained ViT 3H**	**Cropping**	0.03	0.95	-0.93	-105.07	0.00	2
**Fig 8**	**Trained ViT 3H**	**Gaussian Blur**	0.04	0.93	-0.89	-28.09	0.00	2
**Fig 8**	**Trained ViT 3H**	**Grayscale**	0.72	0.22	0.49	13.98	0.01	2
**Fig 8**	**Trained ViT 3H**	**Horizontal Flip**	0.02	0.97	-0.95	-284.00	0.00	2
**Fig 8**	**Trained ViT 3H**	**No Augmentation**	0.04	0.92	-0.88	-16.63	0.00	2
**Fig 8**	**Trained ViT 6H**	**Color Jitter**	0.81	0.13	0.68	9.85	0.01	2
**Fig 8**	**Trained ViT 6H**	**Cropping**	0.03	0.94	-0.91	-62.40	0.00	2
**Fig 8**	**Trained ViT 6H**	**Gaussian Blur**	0.06	0.89	-0.83	-57.35	0.00	2
**Fig 8**	**Trained ViT 6H**	**Grayscale**	0.79	0.16	0.63	7.50	0.02	2
**Fig 8**	**Trained ViT 6H**	**Horizontal Flip**	0.03	0.94	-0.92	-41.94	0.00	2
**Fig 8**	**Trained ViT 6H**	**No Augmentation**	0.05	0.92	-0.87	-49.51	0.00	2
**Fig 8**	**Trained ViT 9H**	**Color Jitter**	0.85	0.11	0.74	20.52	0.00	2
**Fig 8**	**Trained ViT 9H**	**Cropping**	0.03	0.95	-0.92	-92.00	0.00	2
**Fig 8**	**Trained ViT 9H**	**Gaussian Blur**	0.05	0.89	-0.85	-70.45	0.00	2
**Fig 8**	**Trained ViT 9H**	**Grayscale**	0.74	0.21	0.52	9.99	0.01	2
**Fig 8**	**Trained ViT 9H**	**Horizontal Flip**	0.02	0.96	-0.94	-61.54	0.00	2
**Fig 8**	**Trained ViT 9H**	**No Augmentation**	0.04	0.91	-0.87	-22.98	0.00	2
**Fig 9**	**Trained ViT 1H**	**Fovea Size: 0**	0.30	0.67	-0.37	-18.50	0.00	2
**Fig 9**	**Trained ViT 1H**	**Fovea Size: 7.5**	0.08	0.91	-0.83	-54.34	0.00	2
**Fig 9**	**Trained ViT 1H**	**Fovea Size: 15**	0.01	0.98	NA	NA	NA	NA*
**Fig 9**	**Trained ViT 1H**	**Fovea Size: 30**	0.01	0.99	NA	NA	NA	NA*
**Fig 9**	**Trained ViT 1H**	**Fovea Size: 60**	0.01	0.98	-0.97	-84.00	0.00	2
**Fig 9**	**Trained ViT 1H**	**Fovea Size: 120**	0.03	0.93	-0.90	-155.88	0.00	2
**Fig 9**	**Trained ViT 3H**	**Fovea Size: 0**	0.63	0.34	0.29	12.57	0.01	2
**Fig 9**	**Trained ViT 3H**	**Fovea Size: 7.5**	0.64	0.33	0.32	5.77	0.03	2
**Fig 9**	**Trained ViT 3H**	**Fovea Size: 15**	0.30	0.69	-0.39	-4.10	0.05	2
**Fig 9**	**Trained ViT 3H**	**Fovea Size: 30**	0.01	0.99	-0.98	-146.50	0.00	2
**Fig 9**	**Trained ViT 3H**	**Fovea Size: 60**	0.01	0.98	-0.97	-168.01	0.00	2
**Fig 9**	**Trained ViT 3H**	**Fovea Size: 120**	0.04	0.94	-0.90	-34.44	0.00	2
**Fig 9**	**Trained ViT 6H**	**Fovea Size: 0**	0.72	0.23	0.50	7.37	0.02	2
**Fig 9**	**Trained ViT 6H**	**Fovea Size: 7.5**	0.61	0.35	0.26	2.07	0.17	2
**Fig 9**	**Trained ViT 6H**	**Fovea Size: 15**	0.26	0.72	-0.45	-5.23	0.03	2
**Fig 9**	**Trained ViT 6H**	**Fovea Size: 30**	0.02	0.98	-0.96	-109.23	0.00	2
**Fig 9**	**Trained ViT 6H**	**Fovea Size: 60**	0.01	0.99	NA	NA	NA	NA*
**Fig 9**	**Trained ViT 6H**	**Fovea Size: 120**	0.08	0.86	-0.77	-22.22	0.00	2
**Fig 9**	**Trained ViT 9H**	**Fovea Size: 0**	0.79	0.17	0.62	9.93	0.01	2
**Fig 9**	**Trained ViT 9H**	**Fovea Size: 7.5**	0.71	0.24	0.47	6.01	0.03	2
**Fig 9**	**Trained ViT 9H**	**Fovea Size: 15**	0.42	0.55	-0.13	-0.61	0.60	2
**Fig 9**	**Trained ViT 9H**	**Fovea Size: 30**	0.02	0.97	-0.95	-65.15	0.00	2
**Fig 9**	**Trained ViT 9H**	**Fovea Size: 60**	0.01	0.98	-0.98	-110.74	0.00	2
**Fig 9**	**Trained ViT 9H**	**Fovea Size: 120**	0.06	0.89	-0.82	-49.40	0.00	2
**Fig 10**	**Trained ViT 3H**	**Test Set 1 (Visual Binding Shapes)**	0.44	0.44	0.01	0.08	0.94	5
**Fig 10**	**Trained ViT 3H**	**Test Set 2 (Basic Shapes)**	0.47	0.23	0.24	5.13	0.00	5
**Fig 10**	**Trained ViT 3H**	**Test Set 3 (Realistic Shapes)**	0.60	0.18	0.43	12.56	0.00	5
**Fig 10**	**Untrained ViT 3H**	**Test Set 1 (Visual Binding Shapes)**	0.07	0.90	-0.83	-39.87	0.00	2
**Fig 10**	**Untrained ViT 3H**	**Test Set 2 (Basic Shapes)**	0.11	0.83	-0.72	-215.00	0.00	2
**Fig 10**	**Untrained ViT 3H**	**Test Set 3 (Realistic Shapes)**	0.15	0.66	-0.51	-88.33	0.00	2

* NA denotes that the performance between the three seeds was nearly identical, preventing a *t*-test.

**Table 3:** Results of unpaired Welch’s two-sample *t*-tests comparing shape and color scores between trained and untrained models.

			Shape *t*-tests			Color *t*-tests		
Figure	Model Architecture	Training Condition	*t*	*p*	*df*	*t*	*p*	*df*
**Fig 1**	**ViT 1H**		3.58	0.03	3.67	-4.14	0.02	3.17
**Fig 1**	**ViT 3H**		26.23	0.00	2.73	-53.72	0.00	2.31
**Fig 1**	**ViT 6H**		15.84	0.00	2.00	-23.14	0.00	2.05
**Fig 1**	**ViT 9H**		18.33	0.00	2.05	-18.14	0.00	2.04
**Fig 2**	**ViT 1H**		8.88	0.00	3.20	-8.90	0.00	3.63
**Fig 2**	**ViT 3H**		30.64	0.00	2.14	-34.68	0.00	2.25
**Fig 2**	**ViT 6H**		20.49	0.00	2.06	-34.87	0.00	3.74
**Fig 2**	**ViT 9H**		25.81	0.00	2.11	-31.98	0.00	3.67
**Fig 3**	**ViT 6H**		68.51	0.00	2.00	-116.28	0.00	2.94
**Figs 4–6**	**ViT 1H**	**Dense Exploration**	18.38	0.00	2.56	-19.00	0.00	3.20
**Figs 4–6**	**ViT 1H**	**No Depth Transitions**	4.19	0.05	2.05	-9.44	0.01	2.23
**Figs 4–6**	**ViT 1H**	**No Head Movements**	15.01	0.00	3.47	-13.29	0.00	2.38
**Figs 4–6**	**ViT 1H**	**No Side-to-Side Transitions**	2.88	0.10	2.09	-3.85	0.06	2.06
**Figs 4–6**	**ViT 1H**	**No Transitional Views**	3.26	0.08	2.10	-5.30	0.03	2.13
**Figs 4–6**	**ViT 1H**	**Shuffled Images**	0.35	0.75	2.56	0.94	0.40	3.92
**Figs 4–6**	**ViT 3H**	**Dense Exploration**	4.36	0.05	2.02	-25.45	0.00	2.69
**Figs 4–6**	**ViT 3H**	**No Depth Transitions**	41.90	0.00	3.04	-67.35	0.00	2.31
**Figs 4–6**	**ViT 3H**	**No Head Movements**	6.60	0.02	2.06	-25.11	0.00	2.82
**Figs 4–6**	**ViT 3H**	**No Side-to-Side Transitions**	4.79	0.04	2.05	-15.57	0.00	2.33
**Figs 4–6**	**ViT 3H**	**No Transitional Views**	10.18	0.01	2.25	-19.13	0.00	2.50
**Figs 4–6**	**ViT 3H**	**Shuffled Images**	3.41	0.06	2.34	-0.72	0.55	2.12
**Figs 4–6**	**ViT 6H**	**Dense Exploration**	15.01	0.00	2.00	-34.61	0.00	2.08
**Figs 4–6**	**ViT 6H**	**No Depth Transitions**	4.44	0.05	2.00	-15.05	0.00	2.02
**Figs 4–6**	**ViT 6H**	**No Head Movements**	6.55	0.02	2.00	-16.99	0.00	2.03
**Figs 4–6**	**ViT 6H**	**No Side-to-Side Transitions**	4.00	0.06	2.00	-11.20	0.01	2.01
**Figs 4–6**	**ViT 6H**	**No Transitional Views**	22.30	0.00	2.00	-28.43	0.00	2.08
**Figs 4–6**	**ViT 6H**	**Shuffled Images**	2.11	0.17	2.00	-3.07	0.09	2.00
**Figs 4–6**	**ViT 9H**	**Dense Exploration**	44.13	0.00	2.16	-186.68	0.00	4.00
**Figs 4–6**	**ViT 9H**	**No Depth Transitions**	3.30	0.08	2.03	-92.57	0.00	2.94
**Figs 4–6**	**ViT 9H**	**No Head Movements**	4.63	0.04	2.06	-39.00	0.00	2.14
**Figs 4–6**	**ViT 9H**	**No Side-to-Side Transitions**	8.53	0.01	2.19	-21.58	0.00	2.05
**Figs 4–6**	**ViT 9H**	**No Transitional Views**	3.69	0.06	2.05	-11.62	0.01	2.01
**Figs 4–6**	**ViT 9H**	**Shuffled Images**	2.90	0.10	2.02	-2.51	0.13	2.00
**Fig 7**	**SimCLR-CLTT 10L**	**Dense Exploration**	19.47	0.00	2.02	-15.87	0.00	2.12
**Fig 7**	**SimCLR-CLTT 10L**	**No Depth Transitions**	19.52	0.00	2.02	-15.64	0.00	2.19
**Fig 7**	**SimCLR-CLTT 10L**	**No Head Movements**	19.26	0.00	2.04	-16.09	0.00	2.01
**Fig 7**	**SimCLR-CLTT 10L**	**No Side-to-Side Transitions**	18.81	0.00	2.02	-16.00	0.00	2.02
**Fig 7**	**SimCLR-CLTT 10L**	**No Transitional Views**	18.97	0.00	2.09	-16.21	0.00	2.02
**Fig 7**	**SimCLR-CLTT 10L**	**Shuffled Images**	-0.77	0.48	5.13	1.00	0.37	4.63
**Fig 9**	**ViT 1H**	**Fovea Size: 0**	30.00	0.00	2.00	67.00	0.00	2.00
**Fig 9**	**ViT 1H**	**Fovea Size: 7.5**	8.69	0.01	2.00	136.00	0.00	2.00
**Fig 9**	**ViT 1H**	**Fovea Size: 15**	NA	NA	NA	NA	NA	NA*
**Fig 9**	**ViT 1H**	**Fovea Size: 30**	NA	NA	NA	NA	NA	NA*
**Fig 9**	**ViT 1H**	**Fovea Size: 60**	1.73	0.23	2.00	169.74	0.00	2.00
**Fig 9**	**ViT 1H**	**Fovea Size: 120**	NA	NA	NA	161.08	0.00	2.00
**Fig 9**	**ViT 3H**	**Fovea Size: 0**	52.70	0.00	2.00	29.44	0.00	2.00
**Fig 9**	**ViT 3H**	**Fovea Size: 7.5**	24.71	0.00	2.00	11.24	0.01	2.00
**Fig 9**	**ViT 3H**	**Fovea Size: 15**	6.36	0.02	2.00	13.99	0.01	2.00
**Fig 9**	**ViT 3H**	**Fovea Size: 30**	1.73	0.23	2.00	296.00	0.00	2.00
**Fig 9**	**ViT 3H**	**Fovea Size: 60**	NA	NA	NA	169.74	0.00	2.00
**Fig 9**	**ViT 3H**	**Fovea Size: 120**	6.93	0.02	2.00	46.20	0.00	2.00
**Fig 9**	**ViT 6H**	**Fovea Size: 0**	22.03	0.00	2.00	6.51	0.02	2.00
**Fig 9**	**ViT 6H**	**Fovea Size: 7.5**	10.03	0.01	2.00	5.22	0.03	2.00
**Fig 9**	**ViT 6H**	**Fovea Size: 15**	6.08	0.03	2.00	16.54	0.00	2.00
**Fig 9**	**ViT 6H**	**Fovea Size: 30**	5.00	0.04	2.00	169.74	0.00	2.00
**Fig 9**	**ViT 6H**	**Fovea Size: 60**	NA	NA	NA	NA	NA	NA*
**Fig 9**	**ViT 6H**	**Fovea Size: 120**	5.74	0.03	2.00	42.25	0.00	2.00
**Fig 9**	**ViT 9H**	**Fovea Size: 0**	23.96	0.00	2.00	5.63	0.03	2.00
**Fig 9**	**ViT 9H**	**Fovea Size: 7.5**	16.46	0.00	2.00	6.66	0.02	2.00
**Fig 9**	**ViT 9H**	**Fovea Size: 15**	4.04	0.06	2.00	5.01	0.04	2.00
**Fig 9**	**ViT 9H**	**Fovea Size: 30**	2.65	0.12	2.00	168.01	0.00	2.00
**Fig 9**	**ViT 9H**	**Fovea Size: 60**	2.00	0.18	2.00	147.50	0.00	2.00
**Fig 9**	**ViT 9H**	**Fovea Size: 120**	19.00	0.00	2.00	47.78	0.00	2.00
**Fig 10**	**ViT 3H**	**Test Set 1 (Visual Binding Shapes)**	10.25	0.00	6.29	-7.39	0.00	12.92
**Fig 10**	**ViT 3H**	**Test Set 2 (Basic Shapes)**	13.76	0.00	7.09	-12.98	0.00	12.54
**Fig 10**	**ViT 3H**	**Test Set 3 (Realistic Shapes)**	19.58	0.00	7.38	-15.94	0.00	9.91

* NA denotes that the performance between the three seeds was nearly identical, preventing a *t*-test.

## Supporting information

S1 Fig Generalization check. (a) To test whether the results would generalize across objects, we tested the transformers with new colors and shapes. (b) Untrained transformers had color-based representational spaces, as shown in the RDMs (*left*) and color/shape scores (*right*). (c) Trained transformers developed shape perception. The one exception was the smallest (1H) model, which failed to develop shape perception. (d) t-SNE visualizations showed that untrained transformers group objects based on color, whereas (e) trained transformers group objects based on shape. These models were trained on the human adult data from Experiment 1 (UT Austin dataset). For all RDMs, the images used to make the RDMs were the same as those used in Fig 1. Error bars denote standard error for each model across the color cells and shape cells shown in Fig 1b, c.
(TIF)

S2 Fig Generalization check for controlled-rearing experiments. (a) To test whether the results would generalize to other objects, we repeated all of the controlled-rearing conditions with a new object. The results replicated the original pattern: (b) untrained transformers grouped objects by color, whereas (c) trained transformers grouped objects by shape. Transformers largely failed to learn shape perception when (d) the views were shuffled, (e) head movements were ablated, (f) transitional views were ablated, (g) side-to-side transitions were ablated, and (h) depth transitions were ablated. For all RDMs, the images used to make the RDMs were the same as those used in Fig 1. Error bars denote standard error for each model across the color cells and shape cells shown in Fig 1b, c.
(TIF)

S3 Fig Visual learning in non-temporal learning models. We tested two non-temporal fitting models: autoencoders and GreedyInfoMax. The models were trained on egocentric visual experiences from human adults (Fig 1a), matching the visual diet of the temporal fitting models. Both the (a) autoencoders and (b) GreedyInfoMax models largely failed to develop shape perception when given the same training data as temporal models (Fig 1e). (c) After training, the models’ representational spaces were still color-based, akin to the representational spaces of untrained models (Fig 1d). The tSNEs show the representational spaces for untrained and trained GreedyInfoMax models. Error bars denote standard error for each model across the color cells and shape cells shown in Fig 1b, c.
(TIF)

S4 Fig Training transformers with different artificial image augmentations: Generalization Check (1st New Dataset). (a) Transformers trained with no artificial image augmentations developed color-based representational spaces, as shown in the RDMs (*top*) and color/shape scores (*bottom*). (b-d) Likewise, transformers trained with Gaussian blur, horizontal flip, or random cropping developed color-based representational spaces. (e-f) Conversely, transformers trained with color jitter or grayscale developed shape perception. For all RDMs, the images used to make the RDMs were the same as those used in Fig 1. Error bars denote standard error for each model across the color cells and shape cells shown in Fig 1b, c.
(TIF)

S5 Fig Training transformers with different artificial image augmentations: Generalization Check (2nd New Dataset). (a) Transformers trained with no artificial image augmentations developed robust color-based representational spaces, as shown in the RDMs (*top*) and color/shape scores (*bottom*). (b-d) Likewise, transformers trained with Gaussian blur, horizontal flip, or random cropping developed robust color-based representational spaces. (e-f) Conversely, transformers trained with color jitter or grayscale developed robust shape perception. For all RDMs, the object pairs used to make the RDMs were the same as those used in Figs 1 and 2. Error bars denote standard error for each model across the color cells and shape cells shown in Fig 1b, c.
(TIF)
