# Supplementary figures and images for: Computational origins of shape perception
Source: PLoS Comput Biol. 2025 Dec 15;21(12):e1013674. doi: 10.1371/journal.pcbi.1013674 (PMC12704854; doi:10.1371/journal.pcbi.1013674)

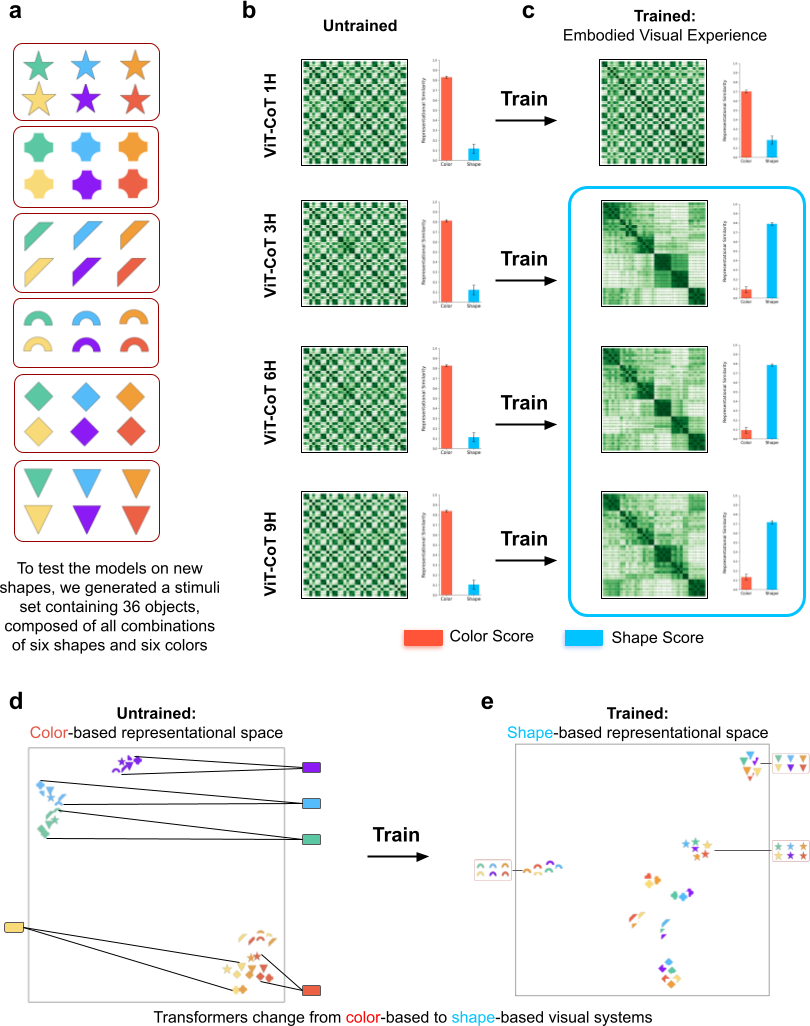

Supplement: S1 Fig — (a) To test whether the results would generalize across objects, we tested the transformers with new colors and shapes. (b) Untrained transformers had color-based representational spaces, as shown in the RDMs (left) and color/shape scores (right). (c) Trained transformers developed shape perception. The one exception was the smallest (1H) model, which failed to develop shape perception. (d) t-SNE visualizations showed that untrained transformers group objects based on color, whereas (e) trained transformers group objects based on shape. These models were trained on the human adult data from Experiment 1 (UT Austin dataset). For all RDMs, the images used to make the RDMs were the same as those used in Fig 1. Error bars denote standard error for each model across the color cells and shape cells shown in Fig 1b, c. (TIF) [file pcbi.1013674.s001.tif]

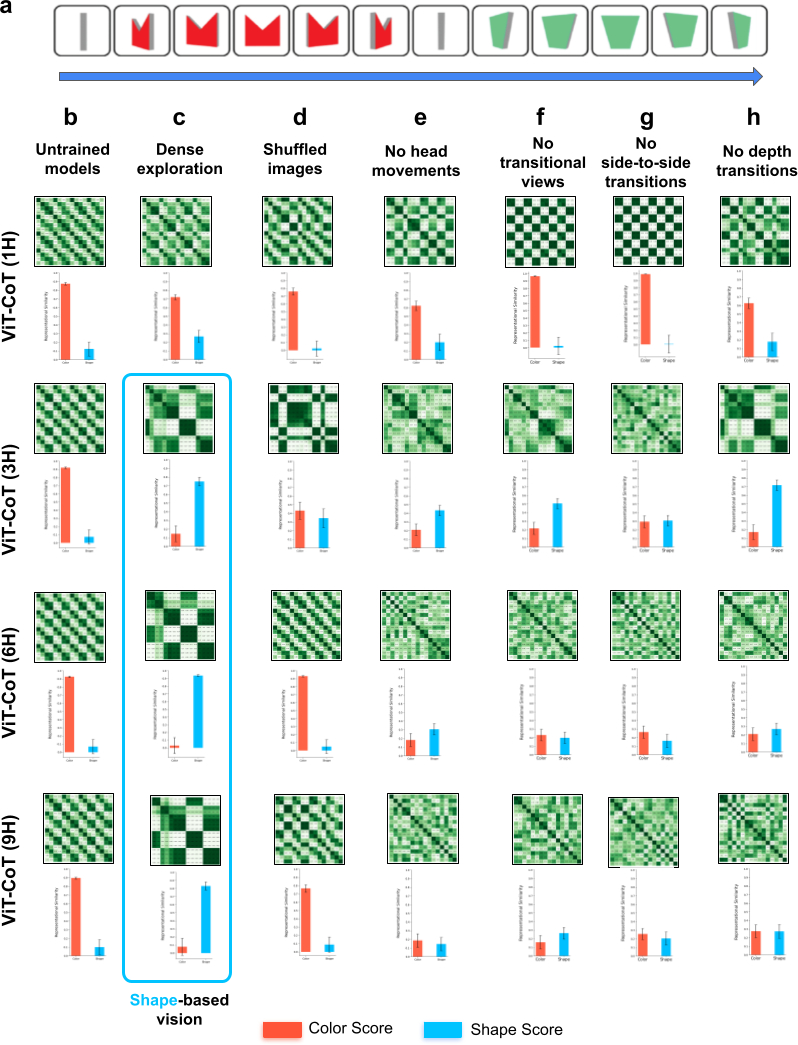

Supplement: S2 Fig — (a) To test whether the results would generalize to other objects, we repeated all of the controlled-rearing conditions with a new object. The results replicated the original pattern: (b) untrained transformers grouped objects by color, whereas (c) trained transformers grouped objects by shape. Transformers largely failed to learn shape perception when (d) the views were shuffled, (e) head movements were ablated, (f) transitional views were ablated, (g) side-to-side transitions were ablated, and (h) depth transitions were ablated. For all RDMs, the images used to make the RDMs were the same as those used in Fig 1. Error bars denote standard error for each model across the color cells and shape cells shown in Fig 1b, c. (TIF) [file pcbi.1013674.s002.tif]

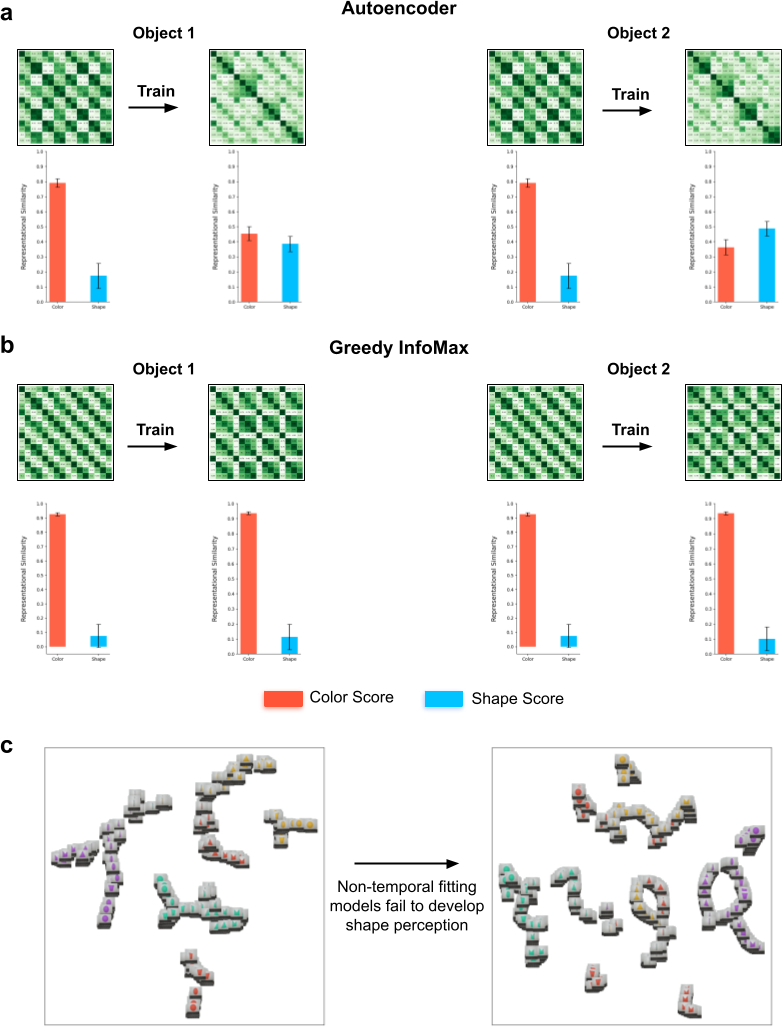

Supplement: S3 Fig — We tested two non-temporal fitting models: autoencoders and GreedyInfoMax. The models were trained on egocentric visual experiences from human adults (Fig 1a), matching the visual diet of the temporal fitting models. Both the (a) autoencoders and (b) GreedyInfoMax models largely failed to develop shape perception when given the same training data as temporal models (Fig 1e). (c) After training, the models’ representational spaces were still color-based, akin to the representational spaces of untrained models (Fig 1d). The tSNEs show the representational spaces for untrained and trained GreedyInfoMax models. Error bars denote standard error for each model across the color cells and shape cells shown in Fig 1b, c. (TIF) [file pcbi.1013674.s003.tif]

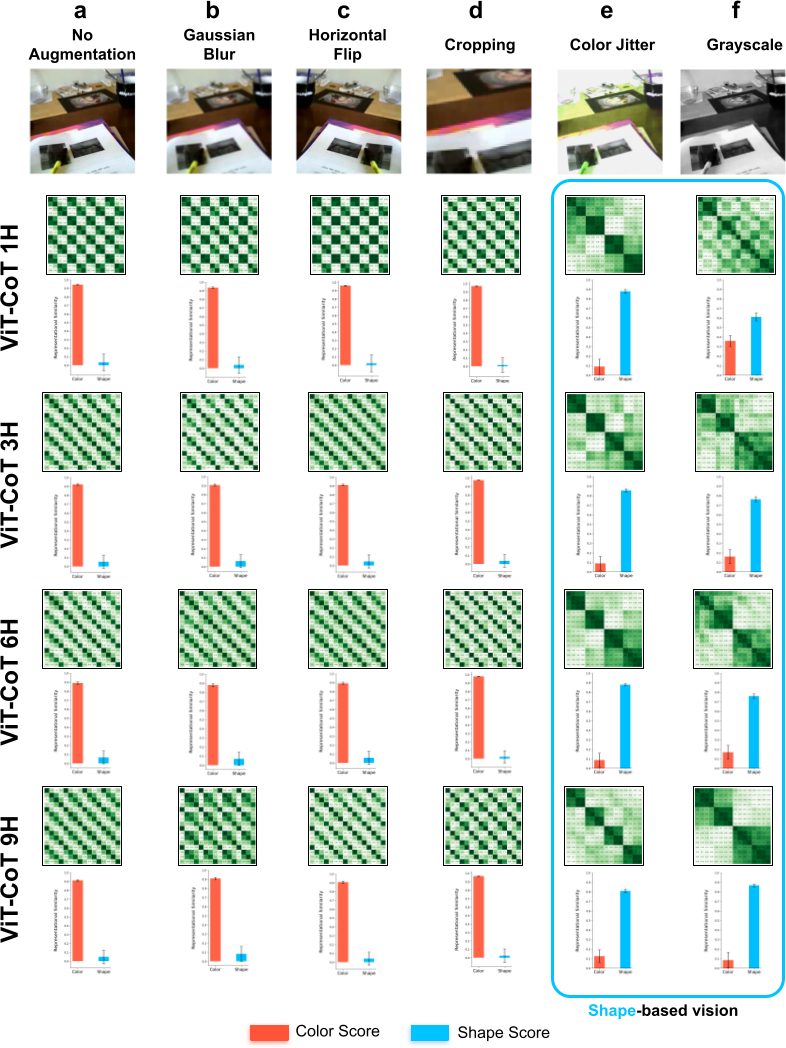

Supplement: S4 Fig — (a) Transformers trained with no artificial image augmentations developed color-based representational spaces, as shown in the RDMs (top) and color/shape scores (bottom). (b-d) Likewise, transformers trained with Gaussian blur, horizontal flip, or random cropping developed color-based representational spaces. (e-f) Conversely, transformers trained with color jitter or grayscale developed shape perception. For all RDMs, the images used to make the RDMs were the same as those used in Fig 1. Error bars denote standard error for each model across the color cells and shape cells shown in Fig 1b, c. (TIF) [file pcbi.1013674.s004.tif]

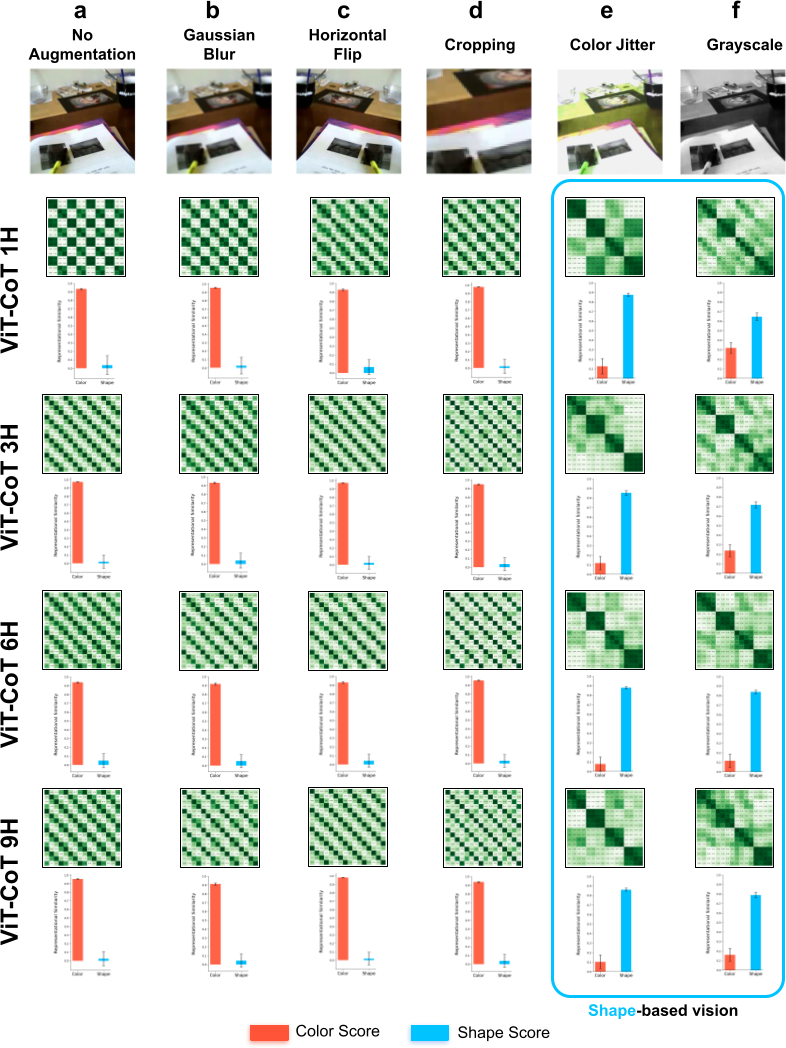

Supplement: S5 Fig — (a) Transformers trained with no artificial image augmentations developed robust color-based representational spaces, as shown in the RDMs (top) and color/shape scores (bottom). (b-d) Likewise, transformers trained with Gaussian blur, horizontal flip, or random cropping developed robust color-based representational spaces. (e-f) Conversely, transformers trained with color jitter or grayscale developed robust shape perception. For all RDMs, the object pairs used to make the RDMs were the same as those used in Figs 1 and 2. Error bars denote standard error for each model across the color cells and shape cells shown in Fig 1b, c. (TIF) [file pcbi.1013674.s005.tif]
